# Triggered or routine site monitoring visits for randomised controlled trials: results of TEMPER, a prospective, matched-pair study

**DOI:** 10.1177/1740774518793379

**Published:** 2018-08-22

**Authors:** Sally P Stenning, William J Cragg, Nicola Joffe, Carlos Diaz-Montana, Rahela Choudhury, Matthew R Sydes, Sarah Meredith

**Affiliations:** MRC Clinical Trials Unit at UCL, University College London, London, UK

**Keywords:** Risk-based monitoring, on-site monitoring, central monitoring, triggered monitoring, targeted monitoring, quality assurance, quality control

## Abstract

**Background/aims:**

In multi-site clinical trials, where trial data and conduct are scrutinised centrally with pre-specified triggers for visits to sites, targeted monitoring may be an efficient way to prioritise on-site monitoring. This approach is widely used in academic trials, but has never been formally evaluated.

**Methods:**

TEMPER assessed the ability of targeted monitoring, as used in three ongoing phase III randomised multi-site oncology trials, to distinguish sites at which higher and lower rates of protocol and/or Good Clinical Practice violations would be found during site visits. Using a prospective, matched-pair design, sites that had been prioritised for visits after having activated ‘triggers’ were matched with a control (‘untriggered’) site, which would not usually have been visited at that time. The paired sites were visited within 4 weeks of each other, and visit findings are recorded and categorised according to the seriousness of the deviation. The primary outcome measure was the proportion of sites with ≥1 ‘Major’ or ‘Critical’ finding not previously identified centrally. The study was powered to detect an absolute difference of ≥30% between triggered and untriggered visits. A sensitivity analysis, recommended by the study’s blinded endpoint review committee, excluded findings related to re-consent. Additional analyses assessed the prognostic value of individual triggers and data from pre-visit questionnaires completed by site and trials unit staff.

**Results:**

In total, 42 matched pairs of visits took place between 2013 and 2016. In the primary analysis, 88.1% of triggered visits had ≥1 new Major/Critical finding, compared to 81.0% of untriggered visits, an absolute difference of 7.1% (95% confidence interval −8.3%, +22.5%; p = 0.365). When re-consent findings were excluded, these figures reduced to 85.7% versus 59.5%, (difference = 26.2%, 95% confidence interval 8.0%, 44.4%; p = 0.007). Individual triggers had modest prognostic value but knowledge of the trial-related activities carried out by site staff may be useful.

**Conclusion:**

Triggered monitoring approaches, as used in these trials, were not sufficiently discriminatory. The rate of Major and Critical findings was higher than anticipated, but the majority related to consent and re-consent with no indication of systemic problems that would impact trial-wide safety issues or integrity of the results in any of the three trials. Sensitivity analyses suggest triggered monitoring may be of potential use, but needs improvement and investigation of further central monitoring triggers is warranted. TEMPER highlights the need to question and evaluate methods in trial conduct, and should inform further developments in this area.

## Introduction

Clinical trial monitoring is defined by the International Conference on Harmonisation (ICH) as ‘The act of overseeing the progress of a clinical trial, and ensuring that it is conducted, recorded and reported in accordance with the protocol, Standard Operating Procedures, Good Clinical Practice (GCP), and the applicable regulatory requirements’, and aims to protect the rights and well-being of trial participants, while ensuring protocol compliance and data integrity.^1^

Monitoring often relies on-site visits, an approach recommended in ICH GCP guidance: ‘In general there is a need for on-site monitoring …’ (section 5.18.3).^1^ Through that guidance, and following high-profile data fraud cases,^2^ on-site monitoring has become a standard means of ensuring GCP compliance since the 1990s, at least in industry-sponsored trials. Visit activities commonly include intensive document review, in particular, source data verification (SDV): the process of checking case report form data against source notes. While this may be done in a sample of patients, or on selected data items on all patients, many trials’ site visits aim to check 100% of trial data.^3^ Intensive on-site monitoring has been highlighted as inefficient and associated with significant costs^4–9^ which are passed down to patients and healthcare systems as drug development expenses.^2,4^

A growing body of evidence shows that 100% SDV is of limited value.^10–13^ Trialists^14,15^ and regulators^16–18^ have expressed support for ‘risk-based monitoring’, recognising that not all clinical trials require the same approach to quality control and assurance. This is reflected in the update of ICH GCP E6.^19^

One risk-based approach is ‘triggered’ or ‘targeted’ on-site monitoring. It was suggested as a possible option for trials of investigational medicinal products classed as low or medium risk by the Medicines and Healthcare products Regulatory Agency, Medical Research Council (MRC), and UK Department of Health.^16^ An initial risk assessment determines the key risks resulting from the intervention and the design of the trial and strategies to minimise those risks are specified. If triggered monitoring is selected, over the course of the trial sites are prioritised for visits based on central monitoring ‘triggers’: predefined indicators such as number of protocol deviations, case report form return rates, uncommon patterns of serious adverse event reporting or subjective assessments of site performance. Such targeted monitoring is also mentioned as a possible approach in the update to ICH GCP E6.^19^

Although triggered monitoring approaches are not uncommon^3^ and have clear potential benefits in terms of resource-use, there is no empirical evidence to show how well they work. TEMPER was designed to provide such evidence.

## Methods

### Study design

**Figure 1.**
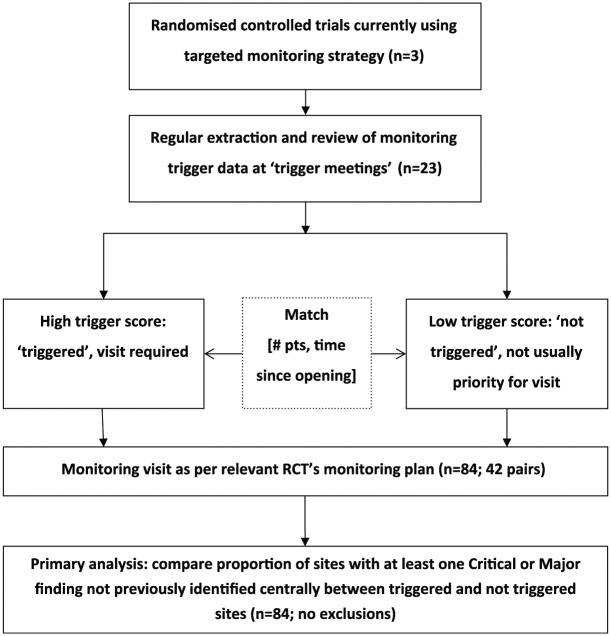
TEMPER study design.

TEMPER is a prospective, matched-pair study assessing the value of triggered monitoring in distinguishing sites with important protocol or GCP compliance issues not identified centrally. Trials unit teams used triggers to identify sites to visit (‘triggered visit’). Each of these was matched with an ‘untriggered site’, and the paired sites were visited and monitored according to the trial’s monitoring plan. Site visit findings were categorised according to a standard classification based on a high-level summary (Table 1). We compared the proportion of triggered and untriggered visits with ≥1 Major/Critical finding not identified through central monitoring or previous visits. The study design is summarised in Figure 1. We developed a bespoke system, the TEMPER Management System (TEMPER-MS) to support implementation of the study.^20^

**Table 1.** Classification of monitoring findings in TEMPER.

Grading	Description
Critical	Findings with potential to have serious impact on patient rights, safety or confidentiality Findings that raise doubt about the accuracy or credibility of key trial data Accumulation of Major findings
Major	Deviations from the trial protocol which may result in some questionable data but without impact on trial results Findings with potential, less serious impact on patient rights, safety or confidentiality Accumulation of Other findings
Other	Errors or deviations that have no important impact on data collection, patient safety or confidentiality

Ethics committee advice deemed no ethical review was required because the additional site visits were within the scope of each trial’s monitoring plan. To ensure visits were arranged and conducted as per normal practice, site staff were not explicitly informed about the TEMPER study or the reason for a monitoring visit.

### Trial selection

Included trials were conducted and monitored by the MRC Clinical Trials Unit at University College London; sponsored by the UK MRC; employing a triggered monitoring strategy; investigational medicinal products risk category B (‘somewhat higher risk than standard medical care’) according to MRC/Department of Health/Medicines and Healthcare products Regulatory Agency risk classification.^16^ Trials also needed to have started recruitment before 2012 and plan follow-up to continue until after 2014.

### Triggers

Triggers were based on those in use in the selected trials, with some additional quantification where thresholds of concern had not previously been defined. The triggers were mainly quantitative although subjective ‘general concerns’ could also be added to each site’s overall trigger score in response to, for example, worrying contact with a site or other more objective concerns not captured by the trial’s triggers. As risks and monitoring needs changed over time, some new triggers were added and/or thresholds modified (e.g. one trial demanded a higher threshold for data return ahead of an interim analysis). Table 2 summarises and exemplifies the trigger types used by trial at the completion of TEMPER.

**Table 2.** Trigger types used during the course of TEMPER by trial.

Trigger type	Description/example	Total triggers per type per trial		
		Trial 1	Trial 2	Trial 3
General concern	Subjective assessment of site performance and/or objective concerns not covered by triggers	1	1	1
Overall CRF return rate	Eg < 80% of expected CRFs received + >20 CRFs outstanding	1	1	1
Return rate, specific CRF	As above, for specific CRF	0	0	3
Return rate, Patient consent form	As above for specific CRF	0	0	1
Data query rate (overall)	Eg > 5% of data items missing or under query	1	1	1
Data query rate (specific question)	As above, for specific data item	1	0	0
Data query resolution time	Eg > 50% of missing or queried data items outstanding for >3 months	1	1	1
SAE rate (high)	Eg number SAEs/person years on study > threshold (based on average for trial)	1	0	1
SAE rate (low)	Eg number SAEs/person years on study < threshold (based on average for trial)	0	1	1
Protocol deviation (treatment)	Eg treatment administered when clinical tests out of range	1	9	0
Protocol deviation (eligibility)	Eg date of investigation out of range	3	0	0
Protocol deviation (procedure)	Eg failure to perform blood test when mandated	0	1	0
Protocol deviation (withdrawal rate)	Eg > 20% of patients at site recorded as completely withdrawn from trial	0	1	0
High recruitment^a^	>30 patients (Trial 1); >10% patients (trial 2 – never met)	1	1	0
Total triggers assessed		11	17	10

CRF: case report form; SAE: serious adverse event.

a For exploratory prognostic analyses, a high recruitment trigger was defined retrospectively for all trials as a site ranked in the top 10% of sites ordered by recruitment.

### Site selection

We scheduled regular ‘trigger meetings’ (3–6 monthly or more frequently if required) with trials unit teams to review trigger data. Sites’ trigger scores were calculated by the TEMPER-MS and reviewed by the trial teams to decide which sites to visit. Chosen sites usually had the highest total trigger scores, but general concerns sometimes led to other sites being prioritised. All trigger meeting discussions were documented.

To replicate real-life prioritisation by resource-limited trial teams, we asked teams to distinguish between sites that would definitely be visited in normal practice (‘triggered-and-usually-visited’); and those, usually with lower trigger scores, considered lower priority for a visit at that time (‘triggered-but-not-usually-visited’); both are grouped as ‘triggered visits’ for the primary analysis.

The TEMPER-MS matching algorithm proposed ‘untriggered’ sites to visit, minimising differences in (1) number of patients and (2) time since first patient randomised, while maximising differences in trigger score (see Appendix in Supplementary Material and Diaz-Montana et al.^20^). The closest match was accepted, unless there were clearly documented reasons not to. For example, an untriggered site that had been visited very recently outwith TEMPER was replaced with the next closest match.

### Site visits

To maximise similarity between triggered and untriggered monitoring visits, they were all conducted according to the trial’s monitoring plan with the same planned checks at all visits in addition to follow-up of any specific concerns raised by the trials unit team. These were broadly similar across the trials in the study: monitoring usually included SDV on a sample of patients and review of consent forms, pharmacy documents and facilities, and Investigator Site Files.

We aimed to conduct all visits within 3 months after the trigger meeting, with paired visits as close together as possible, and no more than 28 days apart. The triggered visit was planned before its untriggered match to help ensure that any changes to monitoring visit approach implemented by the trial team at the time of the triggered visit could be reflected in the paired visit. All monitors performed the same roles at site visits. Triggered visits were attended by TEMPER-specific and trial-specific monitors, untriggered visits only by TEMPER monitors. The same GCP and monitoring training was undertaken both by the trial team members attending visits and the monitors; the latter also received trial-specific training. A TEMPER-specific monitoring visit report ensured consistent reporting. Reports were written and followed-up according to each trial’s monitoring plan and trials unit procedures.

### Data collection, finding classification and endpoint definition

Findings were classified as ‘Critical’, ‘Major’ or ‘Other’ (see Table 1), with their final grade taking account of any relevant response from the site to the monitoring report. All Critical and Major findings were further categorised as new or ‘already known prior to the monitoring visit’ (e.g. through central monitoring or self-reporting by the site). The latter were excluded from the primary outcome, but included in the monitoring report to allow follow-up to resolution by the trial team as required. The protocol provided detailed guidance on appropriate gradings (see Online Supplementary Material). This was updated to incorporate new findings as they arose. Selected findings (related to consent and missed serious adverse events in particular), if repeated to a predefined level, could be ‘upgraded’ from ‘Other’ to ‘Major’ or from ‘Major’ to ‘Critical’. In these cases, one additional finding of the higher grade was added to the total findings for that visit.

### Outcome measures

The primary outcome measure was the proportion of sites with ≥1 Major or Critical finding not already identified through central monitoring or a previous visit (‘new’ findings). Secondary outcomes were number of Major and Critical findings, proportion of sites with ≥ 1 Critical finding, number of Critical findings and category of Major/Critical findings.

### Sample size

Sample size calculations, based on review of previous trials unit monitoring reports from trials using triggered monitoring, assumed ∼70% of triggered visits would produce ≥1 new Major/Critical finding. To detect an absolute reduction of ≥30% (from 70% to 40%) in untriggered sites, with 80% power and two-sided significance level of 5%, required ∼84 site visits in 42 matched pairs. We sought balanced numbers of visits across trials and required ≥50% of each trial’s triggered visits to be ‘triggered and usually visited’. Ten additional, unmatched visits were made to high recruiting sites not otherwise selected for visits, to allow further assessment of ‘high recruitment’ as a predictor of findings in secondary analyses.

### Analysis

The primary analysis was a two-group comparison of the proportion of sites with ≥1 new Major/Critical finding in the triggered versus untriggered groups. Analyses of total numbers of Major and Critical findings used one-sample t-tests of the within-pair differences. Prior to the first analysis, the TEMPER Endpoint Review Committee recommended a sensitivity analysis to exclude all findings related to re-consent, as these typically communicated minor changes in side-effect profile that could have been communicated without requiring re-consent. A second, exploratory analysis, excluded *all* consent-related findings because previous research suggested that these could likely be identified centrally.^21,22^

In secondary analyses, using all 94 visits, and with additional information of potential prognostic value obtained from questionnaires completed by the Trials unit and site staff prior to the monitoring visits (see Online Supplementary Material), the ability of individual triggers and site characteristics to predict on-site findings was assessed by comparing the proportion of visits with the outcome of interest (Eg ≥ 1 Major/Critical finding) at sites where a trigger had/had not fired. This utilised chi-square tests (with trend for ordered categories) or Fisher’s exact test as appropriate for univariate analyses and logistic regression for multivariate analyses.

### Consistency

To reduce intra- and inter-observer bias, in addition to Monitor training and the use of the categorisation system, a Consistency Monitoring Group, comprising trials unit staff from the participating trials’ teams, discussed suitable gradings for findings. The Endpoint Review Committee comprised four experienced trialists from the trials unit with no direct link to the trials, and reviewed, for all visit reports and blind to whether the visit was triggered or untriggered, all Major and Critical findings and a selection of Other findings. They also performed cross-visit reviews of similar sorts of findings to ensure consistency of grading. The categorisation appendix and grading of relevant findings from previous visits were updated if required following Consistency Monitoring Group or Endpoint Review Committee discussions.

## Results

Three trials were included; all randomised, multicentre (>100 sites) cancer treatment trials with a time-to-event outcome measure (recurrence-free or overall survival), planned accrual of >1000 patients and paper-based data collection.

### Site selection and matching

In total, 23 trigger meetings and 84 paired monitoring visits took place between 2013 and 2016 (Figure 1). Triggered and untriggered sites had mean trigger scores of 4.0 (range of 2–6) and 0.8 (range of 0–3), respectively. The matching algorithm gave mean within-pair differences (triggered–untriggered) of -1.4 months (70.1 vs 71.5) in time since first randomisation and +8.5 (49.9 vs 41.4) in patients randomised.

### Visit conduct

Three visits were >1 week outside the 3-month visit window. Five untriggered visits were >28 days after their triggered match, the longest gap being 4 months; the continued suitability of the untriggered match as a control was confirmed at the next trigger meeting. One untriggered visit was before its triggered match because of a short-notice postponement.

The median (interquartile range) number of trials unit staff attending triggered and untriggered visits was 3 (2–3) and 2 (2–2), respectively (Wilcoxon p < 0.01). Visit conduct within pairs was similar in most respects: full Investigator Site File checks were done at 25/42 triggered and 27/42 untriggered visits (p = 0.65), pharmacy facility checks at 25 and 29, respectively (p = 0.36), while the median (interquartile range) number of patients undergoing SDV was 4 (3–5) and 4 (3–5), respectively (paired t-test p = 0.08). However, more consent forms were checked at triggered (median (interquartile range): 44 (27–77)) than untriggered visits (35 (18–70)) (paired t-test p = 0.01).

### Primary outcome: Major/Critical findings

Table 3 summarises all Major and Critical findings, and Table 4 summarises the primary outcome; 88.1% of triggered visits had ≥1 new Major/Critical finding, compared to 81.0% of untriggered visits, an absolute difference of 7.1% (95% confidence interval (CI) –8.3%, +22.5%; p = 0.365). When re-consent findings were excluded, these figures reduced to 85.7% versus 59.5% (difference = 26.2% (95% CI 8.0%, 44.4%; p = 0.007)); while excluding *all* consent and re-consent findings reduced them further to 69.0% versus 45.2% (difference = 23.8% (95% CI 3.3%, 44.4%; p = 0.027)). Findings by trial are summarised in Table S1 in the Online Supplementary Material.

**Table 3.** Summary of Major and Critical findings at TEMPER monitoring visits.

Type of finding by monitoring report section	Number of findings^a^				No. (%) sites with ≥ 1Major/Critical finding^b^
	Major		Critical		
	At presentation^c^	Upgradeonly^d^	At presentation	Upgradeonly	
Investigator Site File – All	6	0	0	0	6 (7)
Informed consent – All	219	13	3	12	49 (58)
Re-consent (Eg failure to obtain re-consent in a timely manner)	162	0	0	9	
Original consent (Eg missing signatures, missing or incompatible signature dates, incorrectversions used)	57	13	3	3	
Pharmacy – All	6	0	2	0	5 (6)
CRF/SDV – All	67	3	9	8	43 (51)
Unreported SAE/notable event	25	0	0	4	
Unreported endpoint	12	0	0	4	
Source/priority data discrepancy	19	1	0	0	
Other	11	2	9	0	
Total Major and Critical findings	298	16	14	20	71 (85)

CRF: case report form; SDV: source data verification; SAE: serious adverse event.

a All visits (n = 94).

b Paired visits only (n = 84).

c ‘At presentation’ refers to findings attracting a Major or Critical grade on their own.

d ‘Upgrade only’ refers to groups of findings from the same visit that, collectively, warranted a higher grade (e.g. a series of Major findings at the same site could, in some circumstances, be upgraded to one additional Critical finding).

**Table 4.** Primary and secondary binary outcomes.

	Triggered		Untriggered		Between-group difference (95% CI)	Chi-square test p value
	N	%	N	%		
≥1 Major or Critical finding						
All findings	37	88	34	81	7% (–8%, 23%)	0.365
Excluding re-consent findings	36	86	25	60	26% (8%, 44%)	0.007*
Excluding all consent findings	29	69	19	45	24% (3%, 44%)	0.027*
≥1 Critical finding						
All Findings	15	36	8	19	17% (–2%, 35%)	0.087
Excluding re-consent findings	12	29	5	12	17% (0%, 34%)	0.057
Excluding all consent findings	10	24	5	12	12% (–4%, 28%)	0.150

CI: confidence interval.

* p values ≤ 0.05.

### Secondary outcomes

Critical findings (Online Supplementary Material Table S2) were almost solely from consent form and source data reviews. The majority (59%) were upgrades because of a cumulative number of Major findings, and the remainder were graded Critical in their own right. The proportion of visits with Critical findings was approximately halved in untriggered visits, but these differences were of borderline statistical significance (Table 4).

The median number of new Major and Critical findings (Table 5) was three at triggered visits and one at untriggered visits; the mean within-pair difference was 1.40 (95% CI –0.72, 3.53; p = 0.19) for all findings, 1.05 (95% CI 0.032, 2.06; p = 0.044) excluding re-consent findings and 0.48 (95% CI –0.12, 1.08; p = 0.12) excluding all consent findings (when the median number of findings was 1 and 0, respectively). The median number of new Critical findings was zero at all visits.

**Table 5.** Secondary continuous outcomes.

	Triggered Median (range)	Untriggered Median (range)	Mean within-pairdifference (95% CI)	One-sample t-testp value
Total Major and Critical findings				
All findings	3 (0–24)	1 (0–33)	1.4 (–0.72, 3.53)	0.190
Excluding re-consent findings	1.5 (0–14)	0 (0–6)	1.05 (0.032, 2.06)	0.044*
Excluding all consent findings	1 (0–6)	0 (0–6)	0.48 (–0.12, 1.08)	0.120
Total Critical findings				
All findings	0 (0–5)	0 (0–2)	0.29 (–0.054, 0.62)	0.096
Excluding re-consent findings	0 (0–5)	0 (0–1)	0.29 (–0.02, 0.59)	0.063
Excluding all consent findings	0 (0–2)	0 (0–1)	0.14 (–0.059, 0.34)	0.160

CI: confidence interval.

* p values ≤ 0.05.

### Prognostic value of individual triggers

The ability of specific triggers to predict the presence of Major and/or Critical findings at the site visit was assessed across all outcomes (Online Supplementary Material Tables S3 and S4). While the finding rates tended to be higher when the trigger had been fired at the time of site selection, only three triggers showed even a modest association with outcome (p < 0.05 for at least one outcome, no adjustment for multiple testing). These were ‘data query resolution time’, ‘protocol deviation’ and ‘general concern’. Multivariate analyses were carried out for each outcome measure, but resulted in univariate models only, namely, the trigger with the strongest association with that outcome measure in the univariate analysis.

High-recruiting sites were defined as the top 10% of trial sites ordered by recruitment at the time of the site visit. The prognostic value of high recruitment on outcomes was investigated excluding all consent findings, as the number of consent forms checked was directly related to number of patients. We found no evidence of higher finding rates at these sites.

### Other site characteristics

Trials unit teams completed 90/94 pre-visit questionnaires. There was no clear evidence of a linear relationship between the trial team ratings and the presence of Major or Critical findings, including or excluding consent findings (data not shown).

Pre-visit site questionnaires were provided by 76/94 sites. There was no evidence of a linear association between the chance of ≥1 Major/Critical finding and the number of active trials either per site or per staff member. There was, however, evidence that the greater the number of different trial roles undertaken by the Research Nurse, the lower the probability of Major/Critical findings. To a lesser extent, the reverse was true for the principal investigator (see Online Supplementary Material Table S5).

## Discussion

We have shown that triggered monitoring, as used in these trials, did not satisfactorily distinguish sites with higher and lower levels of concerning on-site monitoring findings. The pre-specified primary comparison showed no significant difference between triggered and untriggered visits in the proportion with ≥1 Major/Critical finding not previously identified centrally. However, over 70% of on-site findings related to issues in recording informed consent, and 70% of these to re-consent; the pre-specified sensitivity analysis excluding re-consent findings demonstrated a clear difference in event rate. There was some heterogeneity between trials in the primary comparison, but much greater consistency in the sensitivity and secondary analyses. In addition, there was some evidence that the trigger process used could identify sites at increased risk of serious concern: around twice as many triggered visits had ≥1 Critical finding, in the primary and sensitivity analyses. Thus, we would suggest that triggered monitoring has promise, but clearly needs refinement.

The categorisation framework we used is, we believe, similar to those applied by regulators to the same findings. However, these typically identify a finding of importance in relation to an individual patient, when it is only by accumulation that these are likely to have serious impact on the trial as a whole. Risk-based monitoring is not looking for perfection in trial data or conduct, but to detect errors that really matter. We found no visit findings that raised serious issues that would apply across sites, involved serious trial-wide safety issues, or suggested any biases across trial arms which would impact credibility of the trials’ results.

The prevalence of sites with Major and Critical findings was higher than expected, echoing the experience of others.^23^ However, the great majority of our findings, like others’,^24^ related to documenting the consent process, for example, ensuring that correct versions are used, and signatures and dates are present and consistent with the timing of randomisation. The ‘quality by design’ concept^25^ states the first course of action should be preventive; informed consent form templates used by academic clinical trials units should, therefore, be reviewed to see if their design can be improved and completion errors reduced. Timely central monitoring of consent forms with adequate anonymisation^22^ may mitigate the effects of many consent form completion errors, particularly if trial treatment timelines mean that full consent forms – or at least selected items – can be reviewed before randomisation.

Re-consent was usually provoked by updates to drug safety information, of which participants (at least those still on treatment) should be aware. This can be a lengthy process and therefore difficult to monitor centrally. When re-consent is explicitly required, better central monitoring methods are possible, perhaps using site logs with lists of expected visit dates. However, although regulatory guidance is clear that participants must be informed about *significant* trial updates, the method is not specified.^19^ Research Ethics Committees and Institutional Review Boards may prefer formal, documented informed re-consent, but this may not always be necessary. Waiting until the participant’s next trial visit may sometimes be inferior (certainly in terms of speed) to sending an immediate letter to the participants explaining the changes and asking them to contact their site only if they have concerns.

Beyond consent processes, the majority of other findings were identified from SDV activities. A growing body of evidence suggests intensive SDV is often of little benefit to randomised controlled trials, with any discrepancies found having minimal impact on the robustness of trial conclusions.^11,12,26^ SDV for a sample of participants may be sufficient to detect *systematic* problems,^27,28^ and focussing SDV only on key data items may be appropriate and rational.

We carried out exploratory analyses of the prognostic value of individual triggers to see if visits to sites at which a specific trigger had fired were substantially more likely to find Major or Critical findings than visits to sites at which this trigger was not fired. The sample size was sufficient to detect an absolute difference of approximately 30% in finding rates. Some triggers, including high or low serious adverse event rates, were rarely met so their prognostic value could not be assessed. Three triggers were of potential, though still at best modest value, given the multiple outcome measures assessed: the speed of data query resolution, protocol deviations and ‘general concern’. These triggers were not wholly independent, and it was not possible to combine them in a way that improved finding rate discrimination more than our triggered/not triggered visit categorisation. We note that high recruitment and poor case report form return rates, although commonly used as triggers^3^ were not of clear prognostic value.

Analysis of site staffing and workload suggested that the fewer trial responsibilities held by the research nurse, the higher the chance of a Major/Critical finding, with a trend to the converse (mainly when findings relating to consent are excluded), in relation to the principal investigator. These findings suggest that, while an insufficiently supported and possibly overstretched investigator may impact adversely on trial conduct, an experienced, capable local Research Nurse, able to take responsibility for many elements of trial conduct, is key.

Ultimately, the sensitivity and specificity of triggered monitoring depends on the selection of triggers. We found Major and Critical findings at untriggered visits, suggesting it remains necessary to visit these sites unless central monitoring techniques can be improved or the discriminatory value of triggers can increase. We used the trials’ existing triggers – quantified more precisely where needed to facilitate ranking of sites – without any prior assessment of their potential value. The search for more discriminatory triggers should encompass work on Key Performance Indicators^29^ and Central Statistical Monitoring.^30^ Subjective assessments may be of value, but are perhaps more prone to inconsistency, particularly when staff turnover is high, and therefore, harder to generalise. We might also optimise current triggers, for example, with better (non-dichotomous) treatment of continuous variables or greater incorporation of temporal trends.

Planning, conducting and follow-up on monitoring visits is time-consuming and therefore costly,^2,8,9^ so maximising cost-benefit is key. We did not routinely use triggers to guide the content of site visits which was perhaps not optimal. Refined triggers could target specific activities, for example, data quality issues could provoke SDV visits and general concerns could provoke additional training. Prospective study of trigger-defined visits is warranted.

Central monitoring enables review of information across sites and time without the time constraints of a site visit. Maximising these strengths would free more time at visits for targeted SDV and activities best done in-person, for example, process review, building rapport or training.

We acknowledge several limitations. TEMPER was conducted in only three trials of similar type although we see no reason to doubt its applicability to other trials. The trials unit staff present at triggered and untriggered visits were not blind to visit type. TEMPER monitors were at all visits, but trial team staff were only required to attend triggered visits. However, the additional staff at triggered visits often included new trial staff attending for training purposes and the planned activities were the same at all visits. The only notable difference in completed activity within pairs was the number of consent forms checked, which was higher in the triggered visits compared to the untriggered visits. While this could have increased the chance of findings at triggered visits, this appears not to have been the case, the difference in finding rates being greater when consent findings were excluded. Observation bias due to lack of blinding of monitoring staff was mitigated by consistent training on the trials and monitoring methods, the use of a common finding grading system and independent review of all Major and Critical findings which was blind to visit type.

The sample size was modest, but nonetheless adequately powered to detect the minimal differences in visit finding rates necessary to support the triggered monitoring strategy employed in these trials. TEMPER assessed the value of pre-existing triggers, rather than first exploring the best triggers. Evidence to support triggered monitoring comes largely from our sensitivity and exploratory analyses, although these were pre-planned, and recommended by an independent committee, which was blind to visit type.

Research into trial conduct rarely has the rigour we demand of clinical trials. The motivation to study this area comprises (1) the need to monitor trials effectively, minimising risk to patients’ rights and safety and protecting data integrity and (2) the need to do so in a cost-effective manner, noting that monitoring activities are a major component of trial conduct costs at the coordinating centre. TEMPER is one of the few studies to address monitoring strategies in a prospective manner,^23,31,32^ and the first, we believe, to specifically evaluate triggered monitoring. Its results should help challenge and guide the future use of triggered monitoring.

## Supplemental Material

793379_supp_mat – Supplemental material for Triggered or routine site monitoring visits for randomised controlled trials: results of TEMPER, a prospective, matched-pair study Click here for additional data file. Supplemental material, 793379_supp_mat for Triggered or routine site monitoring visits for randomised controlled trials: results of TEMPER, a prospective, matched-pair study by Sally P Stenning, William J Cragg, Nicola Joffe, Carlos Diaz-Montana, Rahela Choudhury, Matthew R Sydes and Sarah Meredith in Clinical Trials

## Supplemental Material

793379_supp_mat_2 – Supplemental material for Triggered or routine site monitoring visits for randomised controlled trials: results of TEMPER, a prospective, matched-pair study Click here for additional data file. Supplemental material, 793379_supp_mat_2 for Triggered or routine site monitoring visits for randomised controlled trials: results of TEMPER, a prospective, matched-pair study by Sally P Stenning, William J Cragg, Nicola Joffe, Carlos Diaz-Montana, Rahela Choudhury, Matthew R Sydes and Sarah Meredith in Clinical Trials

## Supplemental Material

793379_supp_mat_3 – Supplemental material for Triggered or routine site monitoring visits for randomised controlled trials: results of TEMPER, a prospective, matched-pair study Click here for additional data file. Supplemental material, 793379_supp_mat_3 for Triggered or routine site monitoring visits for randomised controlled trials: results of TEMPER, a prospective, matched-pair study by Sally P Stenning, William J Cragg, Nicola Joffe, Carlos Diaz-Montana, Rahela Choudhury, Matthew R Sydes and Sarah Meredith in Clinical Trials

## Supplemental Material

793379_supp_mat_4 – Supplemental material for Triggered or routine site monitoring visits for randomised controlled trials: results of TEMPER, a prospective, matched-pair study Click here for additional data file. Supplemental material, 793379_supp_mat_4 for Triggered or routine site monitoring visits for randomised controlled trials: results of TEMPER, a prospective, matched-pair study by Sally P Stenning, William J Cragg, Nicola Joffe, Carlos Diaz-Montana, Rahela Choudhury, Matthew R Sydes and Sarah Meredith in Clinical Trials

## Supplemental Material

793379_supp_mat_5 – Supplemental material for Triggered or routine site monitoring visits for randomised controlled trials: results of TEMPER, a prospective, matched-pair study Click here for additional data file. Supplemental material, 793379_supp_mat_5 for Triggered or routine site monitoring visits for randomised controlled trials: results of TEMPER, a prospective, matched-pair study by Sally P Stenning, William J Cragg, Nicola Joffe, Carlos Diaz-Montana, Rahela Choudhury, Matthew R Sydes and Sarah Meredith in Clinical Trials

## References

[1] International Conference on Harmonisation of technical requirements for pharmaceuticals for human use (ICH). Guideline for good clinical practice E6(R1), http://www.i ch.org/fileadmin/Public_Web_Site/ICH_Products/Guidelines/Efficacy/E6/E6_R1_Guideline.pdf (1996, accessed 8 September 2017).

[2] Lindblad AS Manukyan Z Purohit-Sheth T et al Central site monitoring: results from a test of accuracy in identifying trials and sites failing food and drug administration inspection. Clin Trials 2014; 11: 205–217. 24296321 10.1177/1740774513508028

[3] Morrison BW Cochran CJ White JG et al Monitoring the quality of conduct of clinical trials: a survey of current practices. Clin Trials 2011; 8: 342–349. 21730082 10.1177/1740774511402703

[4] Yusuf S Bosch J Devereaux PJ et al Sensible guidelines for the conduct of large randomized trials. Clin Trials 2008; 5: 38–39. 18283078 10.1177/1740774507088099

[5] Eisenstein EL Collins R Cracknell BS et al Sensible approaches for reducing clinical trial costs. Clin Trials 2008; 5: 75–84. 18283084 10.1177/1740774507087551

[6] Hearn J Sullivan R. The impact of the ‘Clinical Trials’ directive on the cost and conduct of non-commercial cancer trials in the UK. Eur J Cancer 2007; 43: 8–13. 17118647 10.1016/j.ejca.2006.09.016

[7] Funning S Grahnén A Eriksson K et al Quality assurance within the scope of Good Clinical Practice (GCP)-what is the cost of GCP-related activities? A survey within the Swedish Association of the Pharmaceutical Industry (LIF)’s members. Qual Assur J 2009; 12: 3–7.

[8] Sertkaya A Wong HH Jessup A et al Key cost drivers of pharmaceutical clinical trials in the United States. Clin Trials 2016; 13: 117–126. 26908540 10.1177/1740774515625964

[9] Duley L Antman K Arena J et al Specific barriers to the conduct of randomized trials. Clin Trials 2008; 5: 40–48. 18283079 10.1177/1740774507087704

[10] Andersen JR Byrjalsen I Bihlet A et al Impact of source data verification on data quality in clinical trials: an empirical post hoc analysis of three phase 3 randomized clinical trials. Br J Clin Pharmacol 2015; 79: 660–668. 25327707 10.1111/bcp.12531 PMC4386950

[11] Smith CT Stocken DD Dunn J et al The value of source data verification in a cancer clinical trial. PLoS ONE 2012; 7: 12. 10.1371/journal.pone.0051623 PMC3520949 23251597

[12] Tantsyura V Dunn IM Fendt K et al Risk-based monitoring: a closer statistical look at source document verification, queries, study size effects, and data quality. Ther Innov Regul Sci 2015; 49: 903–910. 30222374 10.1177/2168479015586001

[13] Sheetz N Wilson B Benedict J et al Evaluating source data verification as a quality control measure in clinical trials. Ther Innov Regul Sci 2014; 48: 671–680. 30227471 10.1177/2168479014554400

[14] Macefield RC Beswick AD Blazeby JM et al A systematic review of on-site monitoring methods for health-care randomised controlled trials. Clin Trials 2013; 10: 104–124. 23345308 10.1177/1740774512467405

[15] Brosteanu O Houben P Ihrig K et al Risk analysis and risk adapted on-site monitoring in noncommercial clinical trials. Clin Trials 2009; 6: 585–596. 19897532 10.1177/1740774509347398

[16] MRC/DH/MHRA Joint Project. Risk-adapted approaches to the management of clinical trials of investigational medicinal products, https://www.gov.uk/gov ernment/uploads/system/uploads/attachment_data/file/34 3677/Risk-adapted_approaches_to_the_management_of_ clinical_trials_of_investigational_medicinal_products.pdf (2011, accessed 8 September 2017).

[17] Food and Drug Administration. Guidance for industry: oversight of clinical investigations – a risk-based approach to monitoring, http://www.fda.gov/downloads/Drugs/…/Guidances/UCM269919.pdf (2013, accessed 8 September 2017).

[18] European Medicines Agency. Reflection paper on risk based quality management in clinical trials, http://www. ema.europa.eu/docs/en_GB/document_library/Scientific_ guideline/2013/11/WC500155491.pdf (2013, accessed 16 January 2017).

[19] International Conference on Harmonisation of Technical Requirements for Pharmaceuticals for Human Use (ICH). Integrated addendum to ICH E6(R1): guideline for good clinical practice E6(R2), http://www.ich.org/fileadmin/Public_Web_Site/ICH_Products/Guidelines/Efficacy/E6/E6_R2__Step_4_2016_1109.pdf (2016, acces-sed 25 February 2017).

[20] Diaz-Montana C Choudhury R Cragg W et al Managing our TEMPER: monitoring triggers and site matching algorithms for defining triggered and control sites in the TEMPER study. Trials 2017; 18: P149. 10.1186/s13063-019-3301-z PMC6471958 30995932

[21] Bakobaki JM Rauchenberger M Joffe N et al The potential for central monitoring techniques to replace on-site monitoring: findings from an international multi-centre clinical trial. Clin Trials 2012; 9: 257–264. 22064687 10.1177/1740774511427325

[22] Journot V Pérusat-Villetorte S Bouyssou C et al Remote preenrollment checking of consent forms to reduce nonconformity. Clin Trials 2013; 10: 449–459. 23529696 10.1177/1740774513480003

[23] Brosteanu O Schwarz G Houben P et al Risk-adapted monitoring is not inferior to extensive on-site monitoring: results of the ADAMON cluster-randomised study. Clin Trials 2017; 14: 584–596. 28786330 10.1177/1740774517724165 PMC5718334

[24] Von Niederhäusern B Orleth A Schädelin S Generating evidence on a risk-based monitoring approach in the academic setting – lessons learned. BMC Med Res Methodol 2017; 17: 26. 28193170 10.1186/s12874-017-0308-6 PMC5307807

[25] Meeker-O’Connell A Glessner C Behm M et al Enhancing clinical evidence by proactively building quality into clinical trials. Clin Trials 2016; 13: 439–444. 27098014 10.1177/1740774516643491 PMC4952025

[26] Olsen R Bihlet AR Kalakou F et al The impact of clinical trial monitoring approaches on data integrity and cost – a review of current literature. Eur J Clin Pharmacol 2016; 72: 399–412. 26729259 10.1007/s00228-015-2004-y

[27] Van den Bor RM Vaessen PW Oosterman BJ et al A computationally simple central monitoring procedure was proposed and effectively applied to empirical trial data with known fraud. J Clin Epidemiol 2017; 87: 59–69. 28412468 10.1016/j.jclinepi.2017.03.018

[28] Grieve AP. Source data verification by statistical sampling: issues in implementation. Drug Inf J 2012; 46: 368–377.

[29] Gough J Wilson B Zerola M et al Defining a central monitoring capability: sharing the experience of TransCelerate BioPharmas approach, part 2. Ther Innov Regul Sci 2016; 50: 8–14. 30236019 10.1177/2168479015618696

[30] Venet D Doffagne E Burzykowski T et al A statistical approach to central monitoring of data quality in clinical trials. Clin Trials 2012; 9: 705–713. 22684241 10.1177/1740774512447898

[31] Journot V Pignon JP Gaultier C et al Validation of a risk-assessment scale and a risk-adapted monitoring plan for academic clinical research studies – the pre-optimon study. Contemp Clin Trials 2011; 32: 16–24. 20951234 10.1016/j.cct.2010.10.001

[32] Hullsiek KH Kagan JM Engen N et al Investigating the efficacy of clinical trial monitoring strategies: design and implementation of the cluster randomized START monitoring substudy. Ther Innov Regul Sci 2015; 49: 225–233. 25973346 10.1177/2168479014555912 PMC4426264

