# Supplementary material for: Triggered or routine site monitoring visits for randomised controlled trials: results of TEMPER, a prospective, matched-pair study
Source: Clin Trials. 2018 Aug 22;15(6):600–9. doi: 10.1177/1740774518793379 (PMC6236642; doi:10.1177/1740774518793379)
Supplement: 793379_supp_mat – Supplemental material for Triggered or routine site monitoring visits for randomised controlled trials: results of TEMPER, a prospective, matched-pair study [file 793379_supp_mat.pdf]

APPENDIX – MATCHING ALGORITHM

We can preliminarily define a "matching score" between two sites, where lower scores mean sites are more similar, as the (Euclidean) distance between their data points in this bi-dimensional space:

$$distance = \sqrt{(x_2 - x_1)^2 + (y_2 - y_1)^2}$$

where ($x_2 - x_1$) is the difference between the two sites in time since first randomisation, and ($y_2 - y_1$) is the difference between the two sites in number of patients.

Although untriggered sites did not have to have a zero trigger score, to be considered by the trial team as an untriggered site (ie one which they would not intend to visit under normal trial circumstances) their score had to be low. In order to prioritise sites with lower scores in the untriggered site selection, i.e. to penalise sites with higher scores, a penalty was added to the distance to complete the matching score definition:

$$matching\ score\ = distance + penalty$$

$$penalty = \ ([site\ score] * p)$$

where *p* is the "penalty" factor that determines the penalty added depending on the candidate site trigger score. For instance, having a triggered site with a trigger score of 4, and two candidate sites A (site score = 0) and B (site score = 2) which both have the same distance from (and therefore are both as similar as) triggered site C, the preferred match would be site A as its matching score would be lower. As the number of triggers assessed in each trial varied, a constant *p* for each participating trial was determined by the TEMPER Statistician (SS), based on testing a range of values of *p* for each trial and a subjective assessment of the adequacy of the matches selected versus the difference in site trigger scores within the pairs during testing. This factor was then applied to all selections in the live study.
