# Supplementary material for: Triggered or routine site monitoring visits for randomised controlled trials: results of TEMPER, a prospective, matched-pair study
Source: Clin Trials. 2018 Aug 22;15(6):600–9. doi: 10.1177/1740774518793379 (PMC6236642; doi:10.1177/1740774518793379)
Supplement: 793379_supp_mat_2 – Supplemental material for Triggered or routine site monitoring visits for randomised controlled trials: results of TEMPER, a prospective, matched-pair study [file 793379_supp_mat_2.pdf]

TEMPER (TargetEd Monitoring: Prospective Evaluation and Refinement)

Prospective evaluation and refinement of a targeted on-site monitoring strategy for multi-centre cancer clinical trials

Version: 3.0
Date: 17 Oct 2013

MRC CTU ID: TEMPER METHODOLOGY HUB TRIAL

Authorised by:

Name:

Role:

GENERAL INFORMATION

This document was constructed using the MRC CTU Protocol Template Version 4.0. It describes the TEMPER study, coordinated by the Medical Research Council (MRC) Clinical Trials Unit (CTU), and provides information about procedures for its conduct. The protocol should not be used as an aide-memoire or guide for the monitoring of other trials. Every care has been taken in drafting this protocol, but corrections or amendments may be necessary.

COMPLIANCE

The study will be conducted in compliance with the study protocol and the individual approved trial protocols, and through them the Declaration of Helsinki, the principles of Good Clinical Practice (GCP), Commission Directive 2005/28/EC with the implementation in national legislation in the UK by Statutory Instrument 2004/1031 and subsequent amendments, the UK Data Protection Act (DPA number: Z5886415), and the National Health Service (NHS) Research Governance Framework for Health and Social Care (RGF).

SPONSOR

The MRC is the study sponsor and has delegated responsibility for the overall management of the TEMPER study to the MRC CTU. The MRC is also the sponsor of the trials included in TEMPER and has delegated responsibility for the overall management of these trials to the MRC CTU. Queries relating to MRC sponsorship of the component trials should be addressed to the Director, MRC CTU, Aviation House, 125 Kingsway, London WC2B 6NH or via the study team.

FUNDING

Cancer Research UK (CRUK) has provided funding for the study (monitors and additional site visits) via grant C1495/A13305 from the Population Research Committee, with additional support for study development and oversight and database development from the MRC London Hub for Trial Methodology Research.

STUDY DETAILS

This study has been registered with the MRC Network of Hubs for Trials Methodology Research . The study protocol will also be submitted to *Trials*, an open access online journal and can be found on the CTU intranet.

STUDY ADMINISTRATION

Please direct all queries to the Clinical Project Manager at MRC CTU in the first instance; trial specific queries will be passed to the Trial Manager of the respective trial.

COORDINATING SITE

MRC Clinical Trials Unit	Switchboard:	020 7670 4700
Methodology Group<br>Aviation House<br>125 Kingsway	Fax:	020 7670 4915
London<br>WC2B 6NH<br>UK	Email:	TEMPER@ctu.mrc.ac.uk

Please refer to individual trial protocols for an up to date list of MRC CTU staff members.

MRC CTU STAFF, TEMPER TRIAL MANAGEMENT GROUP:

**TEMPER Study Monitor:**	Daniel McVeigh	**Tel:**	0207 670 4940
**TEMPER Study Monitor:**	Sue Fleck	**Tel:**	0207 670 4893
**Statistician:**	Sally Stenning	**Tel:**	0207 670 4707
**Senior Analyst Programmer:**	Carlos Díaz-Montaña	**Tel:**	0207 670 4799
**Clinical Project Manager – Data Management Methods:**	Nancy Tappenden	**Tel:**	0207 670 4735
**Clinical Project Manager:**	Nicola Joffe	**Tel:**	0207 670 4793
**Project Leader:**	Sally Stenning	**Tel:**	0207 670 4707
**Chief Investigators:**	Sally Stenning<br>Sarah Meredith	**Tel:**	0207 670 4707<br>0207 670 4787

Please refer to individual trial protocols for an up to date list of MRC CTU staff members.

SUMMARY OF TRIAL

SUMMARY INFORMATION TYPE	SUMMARY DETAILS
**ACRONYM**	TEMPER
**Long Title of Trial**	Prospective evaluation and refinement of a targeted on-site monitoring strategy for multi-centre cancer clinical trials
**Version**	3.0
**Date**	17 October 2013
**MRC CTU ID**	TEMPER Methodology Hub Trial
**Study Design**	The study will use a prospective matched pair design
**Type of Trials to be Studied**	MRC CTU-managed trials funded by CRUK and currently using a targeted monitoring approach
**Interventions to be Compared**	The study will assess the targeted monitoring strategy currently being used at CTU by comparing monitoring findings in targeted vs non-targeted sites.
**Study Hypothesis**	Targeted monitoring identifies sites with a substantially higher rate of major/critical findings at site visits, compared with sites that have not met pre-specified triggers and may therefore be a useful monitoring strategy.
**Primary Outcome Measure(s)**	The proportion of sites with at least one major/critical finding
**Secondary Outcome Measure(s)**	The number of major/critical findings<br>The number/proportion of critical findings<br>The category of major/critical findings, subdivided by<br>Trial-based findings (i.e. issues affecting all patients at a site)<br>Patient-based findings (as a proportion of the number of patients reviewed)
**Randomisation**	Although the trials themselves are randomised, the targeted monitoring study will not be randomised.<br>Matched controls will be used.
**No. sites to be studied**	Up to a maximum of 84 sites in 42 paired visits
**Duration**	Monitoring visits will take place over ~ 2 years
**Ancillary Studies/Substudies**	None
**Funder**	CRUK – additional staffing for the study and equipment<br>MRC Methodology – funding for the database
**Legal Representative in Europe**	MRC
**Project Manager**	Nicola Joffe
**Chief Investigator**	Sally Stenning, Sarah Meredith
**MRC CTU Project Leader**	Sally Stenning

STUDY SCHEMA

Figure 1. Visit/Study Strategy

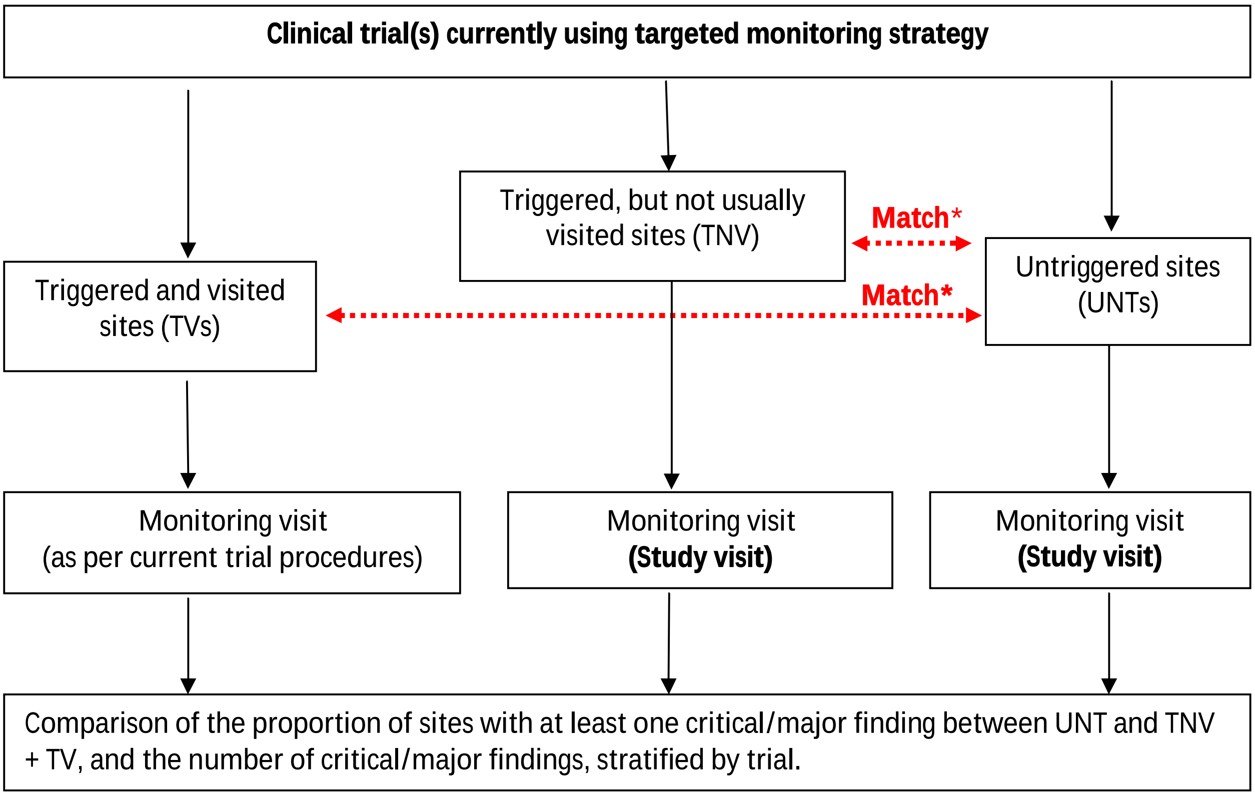

* Sites will be visited within a month of each other where possible, and will be matched as far as possible for both the number of patients enrolled and the time since site approval was given

CONTENTS

GENERAL INFORMATION II

SUMMARY OF TRIAL IV

STUDY SCHEMA 5

CONTENTS 6

ABBREVIATIONS 8

1 BACKGROUND 10

2 SELECTION OF TRIALS 12

2.1 TRIAL INCLUSION CRITERIA 12
2.2 APPROVAL AND ACTIVATION 12
2.3 TRIAL SITE MANAGEMENT 13
3 SITE AND TRIGGER ASSESSMENT 14

3.1 SITE SELECTION 14
3.1.1 Temper Study Visit Definitions 14
3.1.2 Trigger Assessment Process 14
3.1.3 Selection of Matched UNT Sites ..... 15**Error! Bookmark not defined.**
3.1.4 Site Pooling Process 15
3.1.5 Visit Timing 16
3.1.6 Number of Visits 17
4 MONITORING STRATEGY/PROCEDURE 17

4.1 INTRODUCTION 17
4.2 MONITORING STRATEGY 17
4.3 MONITORING PROCEDURE 17
4.3.1 Recording of the Data/Data Collection 17
4.3.2 Categorization of Findings 17
4.3.3 Reporting Mechanisms 18
4.3.4 Follow-up Visits 18
4.3.5 Procedures for Follow-up After the Visit 19
4.4 COMPLIANCE AND ADHERENCE 18
4.5 TRIAL DISCONTINUATION 19
5 REPORTING OF FINDINGS 20

5.1 DEFINITIONS 20
5.1.1 Consistency of findings 20
6 QUALITY ASSURANCE & CONTROL 21

6.1 RISK ASSESSMENT 21
6.1.1 Inter-Observer Bias 21
7 STATISTICAL CONSIDERATIONS 22

7.1 METHOD OF SITE SELECTION 22
7.2 OUTCOME MEASURES 22
7.2.1 Primary Outcome Measure 22
7.2.2 Secondary Outcome Measures 22
7.3 SAMPLE SIZE 22
7.4 INTERIM ANALYSES 23
7.5 ENDPOINT REVIEW 23
7.6 ANALYSIS PLAN (BRIEF) 23
7.6.1 Primary analysis 23

7.6.2 Secondary analyses 24
8 REGULATORY & ETHICAL ISSUES 25
8.1 COMPLIANCE 25
8.1.1 Regulatory Compliance 25
8.1.2 Data Collection and Retention 25
8.2 ETHICAL CONDUCT OF THE STUDY 26
9 INDEMNITY 27
10 FINANCE 28
11 OVERSIGHT & STUDY COMMITTEES 29
11.1 END POINT REVIEW COMMITTEE (ERC) 29
11.2 TRIAL STEERING COMMITTEE (TSC) AND (INDEPENDENT) DATA MONITORING COMMITTEE (I[DMC]) 29
11.3 CONSISTENCY OF MONITORING GROUP (CMG) 29
12 PUBLICATION 30
13 PROTOCOL AMENDMENTS 31
14 REFERENCES 32
APPENDICES 33

ABBREVIATIONS

AE	Adverse Event
AR	Adverse Reaction
CF	Consent Form
CI	Chief Investigator
Ci	Confidence interval
CMG	Consistency of Monitoring Group
CPM	Clinical Project Manager
CRF	Case Report Form
CRUK	Cancer Research United Kingdom
CTU	Clinical Trials Unit
DCF	Data Clarification Form
DM	Data Manager
DMC	Data Monitoring Committee
DPA	(UK) Data Protection Act
DMS	Data Management Systems
DSRT	Data Management Systems Reporting Tool
ERC	Endpoint Review Committee
GCP	Good Clinical Practice
IB	Investigator’s Brochure
ICH	International Conference on Harmonisation of Technical Requirements for Registration of Pharmaceuticals for Human Use
IDMC	Independent Data Monitoring Committee
IMP	Investigational Medicinal Product
MHRA	Medicines and Healthcare products Regulatory Agency
MRC	Medical Research Council
MRC CTU	Medical Research Council Clinical Trials Unit
NCRN	National Cancer Research Network

NHS	National Health Service
PI	Principal Investigator
PRC	Population Research Committee (Part of CRUK – Cancer Research UK)
QA	Quality Assurance
QC	Quality Control
QMP	Quality Management Plan
R&D	Research and Development
RGC	Research Governance Committee
RGF	Research Governance Framework (for Health and Social Care)
SAE	Serious Adverse Event
SAP	Statistical Analysis Plan
SDV	Source Data Verification
SmPC	Summary of Product Characteristics
SOP	Standard Operating Procedure
TSM	TEMPER Specific Monitor
Study	The methodogical study described in this protocol: Prospective evaluation and refinement of a targeted on-site monitoring strategy for multi-centre cancer clinical trials (TEMPER)
TM	Trial Manager
TMF	Trial Master File
TMG	Trial Management Group
TMT	Trial Management Team
TNV	Triggered but Not usually Visited
Trial	Each individual trial whose sites will be monitored as part of this protocol ([redacted])
TSC	Trial Steering Committee
TV	Triggered and Visited
UNT	Untriggered

1 BACKGROUND

Since 2004 clinical trials of medicines in Europe have been required to comply with a regulatory framework based on the principles of Good Clinical Practice (GCP). The objectives of GCP, and of quality control (QC) and assurance (QA) processes in the design and conduct of clinical trials, are to protect the rights and safety of trial participants, and to protect data integrity during the trial. Compliance with GCP as specified by the International Conference on Harmonisation (ICH GCP) requires that the conduct of trials is monitored, but the interpretation of the type, extent and frequency of monitoring activities needed varies greatly. Brosteanu et al (2009) reported from a survey of 17 German medical research networks on monitoring strategies used in non-commercial trials that “the extent of on-site monitoring seemed to depend on the preference of the respective network and on the budget available (or deemed necessary)”. There is increasing concern about the way in which the current regulations (and to some extent their interpretation) have increased clinical trial complexity and cost, such that they have effectively become barriers to the design and conduct of good quality clinical trials (2,3).

Baigent et al. (2008) suggest that a specific misinterpretation of the guidelines has led to a widely held belief that intensive site monitoring, which might not be efficient for identifying the errors that could compromise patient safety or bias study results, is an absolute requirement for compliance with GCP. This has major cost implications for research funders and sponsors as on-site monitoring is one of the highest costing trial activities. They suggest that a risk assessment should guide any monitoring plan and that central statistical techniques should be used to guide the frequency and content of on-site visits, should they be deemed necessary. However, whilst there are published papers describing different types of monitoring and other quality control processes (4, 5, 6), no work has been published in which different monitoring strategies are evaluated and compared in a clinical trial setting, and no evidence that one strategy is more effective than another.

Throughout the clinical trial industry, significant resources are spent on the quality control (QC) and quality assurance (QA) of trial data (3, 7). A pragmatic approach to academic trials is to operate within an overall quality management framework that involves risk assessment, a risk-adapted quality management and monitoring plan that may include both central and on-site monitoring, pro-active data management processes and statistical monitoring methods. When designing the monitoring plan, the nature and complexity of the trial itself, the experience of the staff at clinical sites, and the resources available must all be considered.

Risk-adapted trial monitoring is advocated in the MRC/DH/MHRA 2011 guidance “Risk-adapted approaches to the Management of Clinical Trials of Investigational Medicinal Products”, with triggered monitoring particularly recommended for Type B trials using an IMP associated with a risk to patient safety that is somewhat higher than normal care (8). However, the guidance recognises that there is no evidence that this approach is effective and calls for research into the issue.

Most academic phase III cancer trials in the UK are conducted in the National Cancer Research Network (NCRN). Trials typically have large numbers of participating clinical sites supported by trained research staff, with relatively few patients at any one site. Most trials are testing regimens that use licensed oncology drugs outside their licensed indication. These factors are all important considerations for the risk assessment and the trial monitoring plan.

In this protocol we define the different types of monitoring methods in use as follows:

- **Central data monitoring** - using the trial database at the co-ordinating centre to centrally (i.e. remote from the trial site) monitor data collection, protocol and site process related activities.
- **Other central monitoring** - other coordinating centre activities that provide an insight into how a trial is being run at sites.
- **On-site monitoring** - monitoring activities that take place at a clinical site by the central trial team.
- **Triggered on-site monitoring** - site monitoring that is motivated by pre-defined data or activity-related triggers. In many cases the triggers are combined to prompt a visit, or to prioritise which sites are visited

(Please see Appendix A for further details of the monitoring strategies above)

As public and charitable funds for clinical research are limited, and it is an ethical imperative that such funds are used to improve human health, it is essential that any monitoring strategy is cost effective and efficient. However, in clinical trials, cost efficiency needs to be balanced against the assessment of trial participant safety, and the accuracy and validity of data. Evidence of cost-effective and efficient monitoring strategies would enable more good quality trials to be conducted, leading to the testing of more new treatments or improved treatment strategies to benefit patients.

Triggered monitoring may be a useful tool for QA/QC of trials, particularly academic cancer trials which typically have a large number of sites and limited trial management resources. It is used by around one quarter of academic trial organisations that responded to a recent survey conducted by the Clinical Trials Transformation Initiative (9), and the triggers noted in Appendix A are typical of those used by these organisations. However, since no formal assessment of triggered monitoring has previously been undertaken, as part of the programme of research related to trial conduct within the MRC CTU Hub for Trial Methodology, we propose to conduct a prospective evaluation of a targeted monitoring strategy in UK cancer trials currently being run at MRC CTU.

The study, which will use a prospective matched pair design, will require trial teams to identify triggers using central monitoring techniques and prioritise sites for monitoring visits using the triggers as per normal practice. When a site is identifed for a triggered visit, we will match the site to another taking part in the same trial (matching based on the number of patients recruited and the time since site approval was given to open the tria) that has not been triggered at that time and therefore would not normally be visited. We will then carry out monitoring visits to both the triggered site and its matched control, and compare the proportion of sites with major or critical findings identified at the visit in the triggered sites and the control sites. With this design we hope to determine whether or not targetted monitoring is effective in distinguishing sites where unidentified problems seriously affecting patient rights and safety and/or data integrity are more likely to be found on visiting, and those with a substantially lower chance of identifying such issues.

2 SELECTION OF TRIALS

Overall, the study will evaluate the monitoring strategy in cancer trials currently being conducted by the MRC Clinical Trials Unit and funded by CRUK. The trials will all have applicable regulatory, ethics and R&D approvals.

2.1 TRIAL INCLUSION CRITERIA

To participate in the TEMPER study, the trial must fulfil a set of basic criteria that have been agreed by the TEMPER Trial Management Group (TMG) and are defined below.

- **CRUK FUNDING**

  The trial is funded by CRUK.

- **CURRENT MONITORING STRATEGY**

  It uses a targeted monitoring strategy.

- **TRIAL TIMELINES**

  It started randomisation of patients prior to Jan-2012 and will be continuing to follow-up patients until beyond Dec-2014.

- **SITES**

  It has a large number of UK sites which the MRC is responsible for monitoring.

- **RISK**

  The trial is classified as Type B (IMP use associated with a risk to patient safety that is somewhat higher than the risk of standard medical care) according to the MRC/DH/MHRA risk classification system.

  There will be no reduction in the level of monitoring (central or on-site) for any trial compared to current levels, and this study will therefore not remove any safety monitoring for the participants in these trials.

If for any reason the trial team and/or the Research Governance Committee (RGC) judge that a triggered monitoring approach is no longer appropriate to any of the participating trials, e.g. if the risk category were to increase, the trial would then be discontinued from the study and would be replaced by another eligible trial.
In this case, all outstanding monitoring reports would be followed-up to completion according to the standard study and trial procedures, and the findings from these reports would be included in the analysis as originally planned.

2.2 APPROVAL AND ACTIVATION

The TEMPER Chief Investigators(CIs), Project Lead (PL) and Clinical Project Manager (CPM) have overall responsibility for trial selection for the TEMPER study. Once a trial has been identified as meeting the inclusion criteria, the TEMPER study team will provide the trial team with a copy of this protocol and associated documents.

Trials will only be included in TEMPER with the approval of their Trial Management Team (TMT). Approval for additional trials to be part of this study will be obtained in writing from the trial's PL.

At the time of writing, the following trials are to be included in TEMPER:

[redacted]

Other trials may be recruited, if required.

The number of sites and target number of participants at the time of writing are shown in table 1:

Table 1 Information about participating trials

TRIAL NAME	[redacted]	[redacted]	[redacted]
Date trial opened (first patient enrolled)	[redacted]	[redacted]	[redacted]
Approximate date trial due to end recruitment but continue in follow-up	[redacted]	[redacted]	[redacted]
Number of UK sites	[redacted]	[redacted]	[redacted]
Number of patients expected (total)	[redacted]	[redacted]	[redacted]

2.3 TRIAL SITE MANAGEMENT

All staff at participating trial sites will have received training on their respective trial protocols at site initiation, and on-going training is provided via investigator meetings and/or teleconferences as appropriate.

Sites are monitored remotely by MRC CTU using a variety of central monitoring methods. The Trial Manager(s) and Data Manager(s) also keep in close contact with the sites via email and telephone, and through various periodic communications, including queries and reports generated from the trial database.

Triggers for site vists are identified from central monitoring as per the trial specific monitoring plan.

3 SITE AND TRIGGER ASSESSMENT

3.1 SITE SELECTION

In order to assess current practice, the trial managers of the participating trials and the TEMPER specific monitors (TSM) will continue to follow their existing trial specific monitoring plans. The triggers will be assessed and monitoring visits performed according to the current monitoring plan for each trial, aided by the Data Management Systems Reporting Tool (DSRT) for TEMPER. This will draw trigger data from appropriate sources including the trial database and manual triggers provided by the trial teams, and summarise them according to the number and type of triggers met at that time.

The trial-specific criteria used will be based on the current triggering strategy for the trial (see Appendix B) which typically includes such criteria as:

- concern by the Chief Investigator or Trial Management Group (TMG) or TMT after review of central monitoring findings
- identification of potential serious breach of the protocol or GCP
- extremes (low/high) of recruitment compared to other similar trial sites
- extremes (low/high) of SAE reports compared to other trial sites
- centres that have poor compliance with trial procedures (including CRF return and inappropriate drug administration)
- centres with unusually low return of patient consent forms
- centres with a large amount of missing data or data queries that remain unresolved

Not all of these triggers have objective metrics. Trial teams commonly use the number and potential seriousness of the issues identified and comparison between results from multiple sites to prioritise site visits, and this process will continue in order to identify sites to be visited.

Although high recruitment is a trigger used in several of the TEMPER trials, sites meeting this trigger **only** will be handled differently, refer to section 3.1.2 for details.

3.1.1 TEMPER STUDY VISIT DEFINITIONS

Sites that are selected for monitoring visits as a result of meeting a number of triggers during the trigger assessment will be called “triggered and visited sites” (TV). These will comprise those sites that the trial team would have prioritised for a site visit without TEMPER.

Sites meeting a lower number of triggers than the TV sites and therefore considered of lower priority for a visit - will be called ‘triggered but not usually visited sites’ (TNV) for the purposes of the TEMPER study.

Sites that would not be considered for a site visit based on triggers at that time point will be termed “untriggered” (UNT) sites.

3.1.2 TRIGGER ASSESSMENT PROCESS

Trial specific ‘triggering’ meetings will be held on a 3-monthly basis to assess each active trial site and categorise them at that time point as either potentially TV, TNV or UNT sites. Ad hoc triggering meetings may also take place, if for example a serious concern is raised about a site between these meetings as the result of a suspected serious breach, and an urgent visit planned as part of standard trial processes.

For each TV and TNV site selected for a visit at that time, the closest matching UNT site will be selected by the TEMPER database from the pool of UNT sites for that trial that meet the following criteria:

- Not previously visited as UNT site
- Is in the UK

UNT sites will be matched with TV/TNV sites from the same trial using an algorithm that finds the closest match based on

- Number of patients recruited to the trial
- Length of recruitment to the trial

While minimising the number of triggers met by the matched site.

High recruitment is a trigger used in several of the TEMPER trials. Sites meeting this trigger only will be handled differently because (a) it would not be possible to match these sites based on recruitment and (b) any site performance issues however minor could have a substantial impact simply because of the large numbers of patients potentially affected. Therefore, a sample of sites triggered only on the basis of recruitment numbers will be visited and the TEMPER monitoring report completed, but they will not be matched. Data from these visits will be used in a secondary analysis looking at the prognostic value of triggers for identifying problem sites.

3.1.3 SITE POOLING PROCESS

At the time that the TEMPER study starts, all trial sites in a given trial will be in a monitoring assessment pool, whether or not previous site visits for that trial have taken place. Sites that require follow-up of unresolved issues from a previous monitoring visit will be excluded from the pool.

Sites assessed and visited as TV or TNV in TEMPER will be removed from the pool of potential TV/TNV sites for that trial for the remainder of the study. However, if at subsequent triggering meetings these sites do not meet any triggers, they may be considered (and used only once) as a UNT site. Hence the number of sites in the pool will reduce over time. Once a TV or TNV site has been visited for the TEMPER study, any further visits required according to the trial-specific monitoring plan, either newly triggered or follow-up visits, should take place, but the site will not be matched and data from these visits will not be used in the primary analysis of the TEMPER study.

Sites that are visited as matched UNT sites will be removed from the pool of potential UNT sites for that trial for the remainder of the study. However, they will remain in the pool of study sites that are regularly assessed for monitoring triggers; they may become TV or TNV sites at subsequent triggering meetings and would then be matched with a UNT site.

In summary, each site within each trial may be visited at most twice within the TEMPER study: once as a TV or TNV site and once as a UNT site.

3.1.5 VISIT TIMING

Sites will be given notice of the monitoring visit as described in the trial-specific monitoring plan, but will not be told of the specific reason for the visit in order to reduce bias. They will be asked to confirm that the visit date is acceptable, that the required documents will be made available to the monitors and that the relevant staff will be available.

All sites will be visited within the timeframe specified in the trial monitoring plan, but this will be no more than 3 months after the site being identified as TV, TNV or matched UNT at a triggering meeting. The TV or TNV site will always be visited ahead of the matched UNT site, to ensure that the

monitoring of the control site matches that of the triggered site, for example with respect to the number of patient files reviewed. The UNT matched sites will be visited within one month of the TV/TNV site, where possible. The duration of each visit will usually be between 1 and 3 days.

3.1.6 NUMBER OF VISITS

Over the course of the study period, 42 paired visits to triggered sites (TV and TNV) will take place: i.e. 84 site visits overall. In addition, approximately 10 sites that are triggered on the basis of high recruitment only will be visited.

4 MONITORING STRATEGY/PROCEDURE

4.1 INTRODUCTION

All sites will be monitored according to the trial specific monitoring plans and the findings will be recorded on the TEMPER study monitoring report. The items that are monitored may differ between trials due to the nature of the trials included.

4.2 MONITORING STRATEGY

TV sites will be visited by the TEMPER monitors together with the trial team who would usually undertake the on-site monitoring for the trial. TNV and matched UNT monitoring visits will be performed by the TEMPER monitors alone. The monitors will divide the work during the monitoring visit however they see fit for that particular visit. This will depend on the number of monitoring staff at the visit, number of patients currently recruited at the site, the size of the site file, the number of days of the visit, and the number and seriousness of the issues found during the visit.

During the visit all monitors (TEMPER monitors, TMs or DMs) will follow the trial-specific monitoring plan, but will complete the TEMPER specific monitoring report as mentioned above

4.3 MONITORING PROCEDURE

The following areas should be checked, according to each trials' monitoring plan:

- Investigator Site File
- Informed consent
- Pharmacy File
- Source Data Verification (SDV)

Please refer to TEMPER Quality Management Plan and Working Practices (QMP-WP) and trial specific monitoring plans and working practices for details of specific procedures.

4.3.1 DATA COLLECTION AND RECORDING

All data from the study will be stored in the TEMPER database. The database will record details of the sites that reach individual triggers and whether the site is assessed as being TV, TNV or UNT at triggering meetings. The database will also include a screen for recording the monitoring report where data will be recorded during and/or after the visit, enabling a summary monitoring report to be sent to the site.

4.3.2 CATEGORIZATION OF FINDINGS

TMs, DMs and TEMPER monitors will be trained to recognise and categorise the monitoring findings according to the defined categorisation scheme shown Table 5.1. The severity of the individual findings will be reported, even if they are subsequently grouped to comprise a more serious finding because of their frequency. As new findings arise, these will be added to the categorisation scheme after discussion and agreement by the Consistency of Monitoring Group (CMG) to facilitate consistency throughout the study.

4.3.3 REPORTING MECHANISMS

All findings during a visit will be documented in the monitoring report. The monitoring report will be sent to the site after the visit and sites will be asked to complete a corrective action plan for each issue listed on the report.

All major and critical findings and/or serious breaches found at a monitoring visit will be reported to the trial manager of the trial concerned as soon as possible. Notification of the finding will be sent to the trial manager by email, and ensuing discussions will be documented and filed in the trial master file. Any potential serious breach will also be reported to the Research Governance Committee (RGC) by the trial manager as per MRC CTU serious breach SOP 019.

All monitoring findings will be discussed at three-monthly monitoring meetings (see section 11.3 CMG). Consistency of findings will be discussed, together with any trial related issues.

Please see Section 4.3.6 below for timelines of closing out the monitoring report after a visit.

4.3.4 FOLLOW-UP VISITS

Follow-up visits may be performed as necessary according to trial-specific practices; sites will be given at least 4 weeks' notice of the visit. For consistency, wherever possible the same monitors who performed the intial visit will perform the follow-up visit, although they may be accompanied by other members of CTU staff.

4.3.5 PROCEDURES FOR FOLLOW-UP AFTER THE VISIT

Within 10 working days after a visit, the monitor will send a copy of the monitoring report to the site highlighting any critical or major findings. The site will complete the report as required, and should return it to the monitor within a month of its receipt at site. Even though a month will be given for report completion, corrective action plans for critical findings should have been put in place during or shortly after the visit. The monitor will review the report for completeness and accuracy on its return. Any further queries will be returned to the site. Once the report has been completed to the monitor's satisfaction, it can be signed off and filed according to the trial-specific QMP, with one copy sent to site for the Investigator Site File (ISF), and another filed in the trial master file. The TEMPER database will hold electronic copies of all reports.

4.4 COMPLIANCE AND ADHERENCE

Deviation from the planned study monitoring procedure may occur during a site visit as a result of:

- Patient notes not being available
- The monitor not completing all planned checks due to issues found during the visit
- Requested staff members or representatives of specialist services (e.g. a pharmacist) not being available during the visit

Sites will be given advanced notice of a visit as detailed in the trial-specific monitoring plan. Details of any documents required to be made available, as well as staff members who may be needed will be included in the notification to the sites. Confirmation of receipt of this notification will be requested from the site, together with confirmation that documents and staff will be available to the monitor as requested during the visit. Non-compliance with visit requirements will be recorded in the monitoring report and may be included as a monitoring finding; where such a visit is incomplete, we would aim to visit the site again to complete the checks. Where a site visit cannot be arranged within the TEMPER study timelines for purely logistic reasons, sites would either be visited at a later date or may be supplemented for other 'like-for-like' sites, for the purposes of the TEMPER analyses.

However, any such TV site would be followed up outside of the TEMPER study to ensure that they are complying with the trial's monitoring strategy.

4.5 TRIAL DISCONTINUATION

If, for any reason, a triggered monitoring approach is no longer considered appropriate for a particular trial, e.g. the risk of the trial increases, resulting in the trial team and RGC believing that regular site visits are required, the trial would then be discontinued from the study.

For any discontinued trials, any outstanding monitoring reports would be followed up until completion according to normal study and trial procedures, and the findings from these reports would be included in the analysis as originally planned. The trial may then be replaced by another trial, if deemed necessary by the TEMPER TMT to reach the required number of site visits.

5 REPORTING OF FINDINGS

5.1 DEFINITIONS

All findings will be reported using the critical, major, and other categories. These definitions are given in Table 2 . The monitoring report, and consequently the TEMPER database, will also record whether a finding was identified centrally, at a site visit or both. The categorisation of some findings may differ according to the importance of the issue for a particular trial: e.g. pharmacy events defined as 'major' for one trial, but not for another, depending on the characteristics of the IMP and the trial-specific risk assessment. All such variations will be documented on the findings list.

Table 2. : Definitions

TABLE	DEFINITION
Critical findings	Those that impact, or potentially could impact, directly on participant safety or confidentiality, or create serious doubt in the accuracy or credibility of trial data.
Major findings	Include deviations from the protocol that may result in questionable data being obtained, or errors that consist of a number of minor deviations from regulations, suggesting that procedures are not being followed. Any major finding that is not corrected, or that recurs after initial notification, will be raised to critical status.
Other findings	Are errors or deviations from procedures that do not have an important impact on the data that is collected,and/or do not affect participant safety or confidentiality.

5.1.1 CONSISTENCY OF FINDINGS

All the monitors working on this study (TMs, DMs, TEMPER monitors) will receive training in the categorization of findings. At least two people, including at least one TEMPER monitor, will attend each study visit, so that consensus may be reached between the two individuals on any findings that arise. Furthermore, regular meetings will take place between the TEMPER study team who will discuss all the findings for that month, and will regrade findings if appropriate.

A list of findings will be kept and monitors will have access to this list at all times to ensure that the initial grading is consistent with previous similar findings. Should a trial-specific case be made to grade a finding differently, the Endpoint Review Committee would adjudicate. However, should a new finding arise, that is not already on the list, the monitors will discuss the finding and the proposed grading at the three monthly monitoring meetings, and will add the finding and grading to the list, after agreement with the CMG, to inform future visits.

6 QUALITY ASSURANCE & CONTROL

6.1 RISK ASSESSMENT

The Quality Assurance (QA) and Quality Control (QC) considerations of each individual trial, as well as this study, have been based on formal risk assessments, which acknowledge the risks associated with the conduct of the trial and how to address them with QA and QC processes. QA includes all the planned and systematic actions established to ensure the trial is performed and data generated, documented and/or recorded and reported in compliance with the principles of GCP and applicable regulatory requirements. QC includes the operational techniques and activities undertaken within the QA system to verify that the requirements for quality of the trial-related activities are fulfilled. The Risk Assessment for TEMPER has been reviewed by the RGC and has led to the development of a Quality Management Plan and Working Practices (QMP-WP), which will be kept separately.

Trials are only included in the TEMPER study if the RGC has agreed that a targeted monitoring strategy is appropriate for that trial.

6.1.1 INTER-OBSERVER BIAS

Although the categorisation of monitoring findings is clearly defined in this protocol, one cannot exclude inter- and intra-observer variation between monitors and site visits. For consistency, all the monitors will receive the same training, and at least one of the TEMPER monitors will accompany the trial manager and/ or data manager on triggered monitoring visits. Furthermore, the monitors will meet on a regular basis to review and reach consensus on the category of their monitoring findings in order to promote consistency of categorisation. Should findings occur which do not already appear in the predefined list, these will be discussed at the meeting and added to the list as appropriate, following agreement with the CMG. Prior to the final analysis an end-point review committee will also review the monitoring findings blinded to type of site (i.e. TV/TNV/UNT) to ensure consistency of categorisation and agree on which findings are critical. The committee will include senior staff at the MRC CTU who will not be involved with this study on a daily basis.

7 STATISTICAL CONSIDERATIONS

7.1 METHOD OF SITE SELECTION

The study will use a prospective matched pair design. From the start of TEMPER, central monitoring will be used to identify triggers and the trial teams for each trial involved will decide whether or not a monitoring visit is warranted in the usual way, aided by the database reports summarising the status of triggers. At the same time as the triggered site is identified, a site that has not been triggered at that time (UNT) will be matched (in a 1:1 ratio) with the triggered site on the basis of the number of patients recruited and the time since site approval was given to open the trial. This site will also be visited within one month where possible. The visit findings will be recorded as described below.

For triggered sites, records will be kept of the triggering process and the criteria that prompted the visit.

7.2 OUTCOME MEASURES

7.2.1 PRIMARY OUTCOME MEASURE

The primary outcome is the proportion of sites with at least one major or critical finding found at the site during on-site monitoring.

7.2.2 SECONDARY OUTCOME MEASURES

Secondary outcome measures are:

- The number of major and critical findings
- The number and proportion of critical findings
- The category of major/critical findings, subdivided by
  - Trial-based findings (i.e. issues affecting all patients at a site)
  - Patient-based findings

7.3 SAMPLE SIZE

Review of monitoring reports across MRC CTU cancer trials which employ a targeted monitoring strategy, including those we propose to include in this study, showed that approximately 70% of sites visited as a result of a trigger had major or critical findings detected during the TV, mostly relating to unreported SAEs. Sites visited only because of high recruitment were not included in this figure.

To detect an absolute difference of at least 30% in the proportion of sites with a major/critical finding (e.g. from 70% to 40%, or 60% to 30%) with 80% power and a 2-sided significance level of 5% would require approximately 84 sites to be visited in total, i.e. 42 pairs of triggered (TVs+ TNV) and matched (UNT) sites.

In calculating this sample size, triggers based solely on number of patients randomised are excluded. The rationale for this is that we have no reason to expect a higher rate of major findings at such sites and so its inclusion as a trigger could reduce our ability to show a difference in outcome between triggered and untriggered sites. However, we do wish to assess the value of this trigger, since any

failure to comply with study procedures could potentially have a larger impact on the trial as a whole than more serious issues at sites with few trial participants. Therefore, sites meeting this trigger will be visited as per normal practice, and the findings will be included in a subsidiary analysis, as described in the analysis plan.

Table 3 shows the numbers of visits that took place to triggered sites in 2009 for the participating trials:

Table 3 Number of Triggered Site Visits

TRIAL	NUMBER OF VISITS
[redacted]	5
[redacted]	3
[redacted]	2

According to the current strategy, not all sites that meet a single trigger are visited immediately. In this study we will potentially be visiting all sites that are triggered. We therefore feel that it is possible to achieve 42 visits to triggered sites over the two years of the study, as triggered but not visited sites will be included in this number.

7.4 INTERIM ANALYSES

No interim efficacy analyses are planned for this study.

7.5 ENDPOINT REVIEW

An Endpoint Review Committee will meet as necessary, periodically through the course of the study, and once all visits have been performed to evaluate the findings in a blinded fashion. The members of this committee will not be involved in monitoring the trials that are taking part in this study and will evaluate the findings for consistency across trials and monitoring visits. The committee may be facilitated by senior members of the study team who are not involved in the monitoring of the individual trials taking part in this study.

7.6 ANALYSIS PLAN (BRIEF)

The analyses will be described in detail in a full Statistical Analysis Plan (SAP). This section summarises the main issues.

7.6.1 PRIMARY ANALYSIS

The primary analysis will be a comparison of the proportion of sites in the triggered (TVs and TNVs combined) vs. untriggered groups at which major/critical findings were identified, stratified by trial (i.e. differences within trials will be calculated and then combined). The proportions will be presented with individual 95% confidence intervals and a 95% confidence interval for the difference in proportions; the statistical significance of the difference will be assessed using the Mantel-Haenszel test.

The same analysis will be carried out separately amongst the TV vs UNT pairs and the TNV vs UNT pairs. Power to compare differences in these subgroups will be limited, but this will give an indication as to whether the prioritisation process - used informally to decide which of the triggered

sites are visited as a higher priority when resources are limited - distinguishes sites at particularly high risk of major/critical findings.

For the same comparisons, the number of major/critical findings will be compared; the within-pair differences will be calculated first, then averaged across all paired visits and compared using a paired t-test.

7.6.2 SECONDARY ANALYSES

The role of individual triggers will be assessed within a logistic regression model (unit = a study visit, dependent variable=at least one major or critcial finding at that visit) with each trigger as a separate covariate; a forward stepwise modelling approach will be used to determine which triggers, or which combination of triggers are most predictive of major/critical findings at site. The “high recruitment” trigger will also be assessed within this model, increasing the sample size for this analysis.

The above analyses will be repeated with (a) critical findings (b) site-based major/critical findings and (c) individual patient-based findings as the dependent variable in order to determine which triggers are most predictive of the different types of finding.

Other logistical issues which could impact on patient safety and data accuracy will also be assessed as predictors of monitoring findings, in order to ascertain if these issues might be useful triggers to include in targeted monitoring plans. These may include but are not limited to the following types of issue:

- The size of the site in relation to the number of patients in the trial
- How long the research nurse has been in post at the site, and staff turnover of leading staff members at the site for the trial
- How many trials are being run from the site, and by the team at the site
- The involvement of the PI in the trial (in terms of accessibility, involvement, oversight and commitment to the trial)
- The number of trials that the PI is working on

8 REGULATORY & ETHICAL ISSUES

Each trial participating in the TEMPER study will continue to follow their own approved protocol and procedures. This study is methodological in nature, and although the study is based on clinical trials, it is not a clinical trial itself.

The study will evaluate the monitoring strategy in cancer trials currently being conducted by the MRC Clinical Trials Unit and funded by CRUK. These trials have all been approved by both the MHRA and relevant Ethics Committees. There will be no reduction in the level of monitoring (central or on-site) for any trial compared to current levels, and this study will therefore not remove any safety monitoring for the participants in these trials. In fact, this study will increase the amount of clinical monitoring undertaken for each of the selected trials. Members of staff employed specifically for the triggered monitoring study will receive training in the Data Protection Act, and be bound by the confidentiality agreements agreed by all staff at the MRC CTU. Although staff working on this project might see confidential information, including personal identifying information during site visits, they will hold anonymised data only which will be stored on encrypted laptops or password protected desktops as per MRC policy.

All regulatory and ethical issues pertaining to the trials included in the study are covered in their individual protocols and documentation.

8.1 COMPLIANCE

8.1.1 Regulatory Compliance

The trials involved in TEMPER comply with the principles of the Declaration of Helsinki. TEMPER will also be conducted in compliance with the approved protocols of the trials taking part, this protocol, the principles of GCP as laid down by the Commission Directive 2005/28/EC with the implementation in national legislation in the UK by Statutory Instrument 2004/1031 (The Medicines for Human Use [Clinical Trials] Regulations 2004) and subsequent amendments, the UK Data Protection Act (DPA number: Z5886415), and the National Health Service (NHS) Research Governance Framework for Health and Social Care (RGF).

8.1.2 Data Collection and Retention

Data entry will be performed by the monitors either during site visits, or on return to the office once a visit has been completed. One monitor will take the lead for data collection and entry for each visit and will ensure that all data collected is collated in a meaningful manner and entered onto the study database.

The data collected during this study (i.e. original monitoring reports) will be stored with the trial master files for the relevant trial, and will adhere to the archiving policy of the trial concerned. As such the reports will be available to competent authorities in the event of audit or inspection. Copies of the monitoring reports will be sent to the sites to be filed in their investigator site files. The TEMPER database will hold electronic copies of all reports.

8.2 ETHICAL CONDUCT OF THE STUDY

The monitoring that takes place during this study will not be different to the on-site monitoring that occurs usually in the trials concerned and will follow each trials QMP. There are no specific ethical considerations for this study.

9 INDEMNITY

No specific indemnity for this study has been sought, as the monitoring for this study will fall under the indemnity of the individual trials involved. For these trials prior to 1st August 2013 the MRC Policy outlining MRC Indemnity when acting as a Sponsor, Funder or Employer applied:

www.mrc.ac.uk/Utilities/Documentrecord/index.htm?d=MRC005223

From 1st August 2013 the responsibility for safeguarding the study research data moved from the MRC to University College London. However, the security procedures controlling who has access to the data, staff involved in the day to day handling of data, and the conditions under which they are held did not change. The University will also be responsible for providing the clinical trials insurance for the trials and details of this will be sent to the ethics committee overseeing the study and to the local Hospital Trusts.

10 FINANCE

Funding for the salaries of the TEMPER Study Monitors, their equipment and the cost of the travel and subsistence for the monitoring visits for this study has been provided by Cancer Research UK and the details are contained in an agreement between the MRC and CRUK.

Funding for the study database has been provided from MRC core funding for the London Methodology Hub.

The trials that will be involved in this study are also funded by CRUK, and have appropriate agreements in the place with the funding body, the Sponsor and the sites where the trials are taking place.

11 OVERSIGHT & STUDY COMMITTEES

11.1 ENDPOINT REVIEW COMMITTEE (ERC)

The Endpoint Review Committee will meet approximately 6 monthly and/or prior to the final analysis and will review the monitoring findings blind to group (TV, TNV or UNT) to ensure consistency of interpretation and agree on the major and critical findings with particular reference to those which would potentially affect patient safety or trial conclusions. The ERC will include senior staff at the Clinical Trials Unit who are not involved with this study on a daily basis, and will be overseen by the Chief Investigator and Project Lead.

11.2 TRIAL STEERING COMMITTEE (TSC) AND (INDEPENDENT) DATA MONITORING COMMITTEE (I[DMC])

There will be no Trial Steering Committee (TSC) or Independent Data Monitoring Committee (IDMC) for this study, as the study is methodological in nature. The trials taking part in this study however, will continue to have oversight from their TSCs and IDMCs as per their trial specific protocols. Findings from the monitoring visits undertaken during this study may form part of the data shown, or issues raised with these committees.

11.3 CONSISTENCY OF MONITORING GROUP (CMG)

The Consistency Of Monitoring Group (CMG) will be formed comprising of the Trial Manager and/or Data Manager(s) of the trials that take part in the study, the TSMs, and the Clinical Project Manager. The group will meet on a 3-monthly basis to discuss the monitoring findings and reach consensus in consistency in the grading of the findings. Should a new finding arise that was not previously graded, the group will discuss the appropriate grading and add it to the grading template after discussion with the ERC.

12 PUBLICATION

The data from all TEMPER study visits will be combined for analysis; no individual sites will be identified. The results of this study will be analysed, presented at appropriate conferences and written up for publication irrespective of the results. Authors will include the TEMPER study team and representatives of each of the contributing trials; all trial staff contributing to the conduct of the study will be acknowledged.

13 PROTOCOL AMENDMENTS

Summary of changes:

Version	Protocol Dates	Summary of changes from previous version
1.0		Although this protocol was used by the trial teams, it was in draft format and was not formally signed off as it hadn't been reviewed by the protocol review committee
2.0	June 2013 – Oct 2013	Incorporated comments from the protocol review committee
3.0	Oct 2013 -	Correction of typos in protocol v2.0; update of information with regard to MRC CTU at UCL.

14 REFERENCES

1. Brosteanu O, Houben P, Ihrig K, Ohmann C, Paulus U, Pfistner B, Schwarz G, Strenge-Hesse A, Zettelmeyer U. Risk analysis And risk adapted on-site monitoring in non-commercial clinical trials. Clinical Trials 2009; 6: 585-596.

2. Duley L, Antman K, Arena J, Avezum A, Blumenthal M, Bosch J, Chrolavicius S, Li T, Ounpuu S, Perez C, Sleight P, Svard R, Temple R, Tsouderous Y, Yunis C and Yusuf S. Specific barriers to the conduct of randomised trials. Clinical Trials 2008: 5; 40-48.

3. Yusuf S, Bosch J, Devereaux P, Collins R, Baigent C, Granger C, Califf R and Temple R. Sensible guidelines for the conduct of large randomized trials. Clinical Trials 2008: 5; 38-39.

4. Baigent C, Harrell F, Buyse M, Emberson and Altman D. Ensuring trial validity by data quality assurance and diversification of monitoring methods Clinical Trials 2008: 5; 49-55.

5. Pollock BH. Quality Assurance for Interventions in Clinical Trials. Multicentre Data Monitoring, Data Management and Analysis. CANCER Supplement 1994: 74(9) 2647 – 2652

6. Knatterud GL, Rockhold FW, George SL, Barton FB, Davis CE, Fairweather WR, Honohan T, Mowery R, O'Neil R. Guidelines for Quality Assurance in Multicentre Trials: A Position Paper. Controlled Clinical Trials 1998 19:477 – 493

7. Califf R, Karnash S, Woodlief L. Developing Systems for Cost Effective Auditing of Clinical Trials Controlled Clinical Trials 1997; 18: 651-660.

8. MRC/DH/MHRA Joint Project 2011: Risk-adapted Approaches to the Management of Clinical Trials of Investigational Medicinal Products. Available: http://www.mhra.gov.uk/home/groups/l-ctu/documents/websiteresources/con111784.pdf

9. Morrison B, Neaton J, Mitchel J et al. A CTTI survey of current monitoring practices. Clinical Trials 2010: 7(4) p31

APPENDICES

APPENDIX A: FURTHER EXPLANATION OF MONITORING STRATEGIES

CENTRAL MONITORING

Central monitoring (both central data and other monitoring) techniques are used in all trials conducted at the MRC CTU to varying degrees. These include any or all of the following: data checks performed by data managers on receipt of data and during the data entry and verification process; other site management checks performed by trial managers as part of the day to day management of the trials; checks performed prior to Trial Management Group (TMG) meetings as a method of monitoring the activity of the trial overall against triggers and to identify poor performing sites; endpoint and Serious Adverse Event (SAE) review; statistical data checks performed prior to IDMC/TSC meetings; and data cleaning and checking strategies employed prior to database lock.

ON-SITE MONITORING

On-site monitoring by the central team typically involves discussion of trial processes and activity with site teams, a review of the site master file and other trial related paperwork, a review of participant consent forms, source data verification (SDV) of a sample of cases and/or data items, review of endpoint or event data, review of pharmacy and laboratory processes and trial drug accountability, as necessary. Only some trials at CTU have routine on-site monitoring visits and the approach for the selection of sites where this is performed varies for each trial. For example, in some studies all trial sites undergo 100% SDV on site, while in other studies only sites that reach certain triggers are monitored on site.

TRIGGERED MONITORING

Triggered monitoring is the most common strategy for CTU cancer trials, where trials typically involve large numbers of trial sites (greater than 50), and few patients enrolled in the trial at each site. The main triggers for on-site monitoring defined by the trial teams typically include:

- concern by the Chief Investigator (CI)/Trial Management Team (TMT) after review of central monitoring findings
- identification of serious protocol breach
- extremes (low/high) of recruitment compared to other similar trial sites
- extremes (low/high) of SAE reports compared to other trial sites
- centres that have poor compliance with trial procedures (including CRF return and inappropriate drug administration)
- centres with unusually low return of consent forms
- centres with a large amount of missing data or data queries that remain unresolved

APPENDIX B: TRIGGERS FOR THE TRIALS INVOLVED IN THE TEMPER STUDY

[redacted]:

- Concern by the PL/CI or TMG.
- Sites that generate a high volume of data queries which are not readily resolved
- Sites that regularly do not return CRFs or queries within the timelines defined in the Data Management Plan
- Top recruiting sites (that recruit more than 30 patients)

[redacted]:

- Concern by the PL/CI or TMG.
- Sites that generate a high volume of data queries which are not readily resolved
- Sites that regularly do not return CRFs or queries within the timelines defined in the Data Management Plan
- Serious inappropriate drug administration. Doses greater than 110% or less than 90% than the calculated expected dose will be queried via the [redacted] database. Doses that are 150% or more than the expected dose will be notified to the PL and the site will be contacted. A subsequent inappropriate drug administration of the same magnitude will trigger a monitoring visit.

[redacted]:

- Instances that would require a monitoring visit include but are not restricted to the following:
- Poor recruitment; recruitment at each centre is assessed on a regular basis by the TMG, TSC and IDMC, centres with poor recruitment are regularly contacted by the TMT in an attempt to establish the reasons for the poor recruitment. Such centres may be selected for a monitoring visit if it is felt that this would be the best way to address the issues.
- Over or under reporting of SAEs; The numbers of SAEs submitted per centre is assessed by the TMG on a regular basis, given the large number of variables that are involved in the number of SAEs submitted it is not possible to set precise limit on an expected number of SAEs. The TMG will therefore consider these reports and look for major signals of under or over reporting which could prompt a monitoring visit.
- Unusually low return of consent forms; receipt of consent forms is logged on a regular basis during this process the centres with a large amount of missing consent forms can be easily identified, such centres may then be selected for a monitoring visit.
- Unusually low reported event rate; the numbers of events per centre is likely to be quite small during the initial years of the trial and therefore it will be difficult to assess centres for low event reporting event rate until they have been involved in the trial for some time. The event rate is assessed and reported for meetings of the IDMC, from these reports if any centre is found to have a lower then expected event reporting rate then they may be selected for a monitoring visit.
- Large amount of missing data; the amount of missing data per centre is assessed during the regular data management procedures and for TSC and IDMC reports, centres found to be having persistent issues with data return may be selected for a monitoring visit.
- Large amount of data queries; the amount of data queries per centre can be assessed during the regular data management procedures, centres found to be having persistent issues with data queries may be selected for a monitoring visit.
