# Supplementary material for: Triggered or routine site monitoring visits for randomised controlled trials: results of TEMPER, a prospective, matched-pair study
Source: Clin Trials. 2018 Aug 22;15(6):600–9. doi: 10.1177/1740774518793379 (PMC6236642; doi:10.1177/1740774518793379)
Supplement: 793379_supp_mat_3 – Supplemental material for Triggered or routine site monitoring visits for randomised controlled trials: results of TEMPER, a prospective, matched-pair study [file 793379_supp_mat_3.pdf]

Appendix C: Categorisation of critical, major and other monitoring findings

NB this version of Appendix C was finalised after all TEMPER reports had received their final gradings and had been reviewed by the Endpoint Review Committee (ERC). It documents the rationale for the final gradings, as discussed in ERC and Consistency Monitoring Group (CMG) meetings.

CLASSIFICATION OF THE MONITORING FINDINGS	
**Critical**	Critical findings are those that impact, or potentially could impact, directly on participant safety or confidentiality, or create serious doubt in the accuracy or credibility of trial data
**Major**	Major findings include deviations from the protocol that may result in questionable data being obtained or errors that consist of a number of 'other' deviations from regulations, suggesting that procedures are not being followed. Any major finding that is not corrected, or that recurs after initial notification, will be raised to critical status.
**Other**	Other findings are errors or deviations from procedures that do not have an important impact on the data that is collected, or do not affect participant safety or confidentiality.

CODES USED THROUGHOUT	
**REF**	Document is absent from document file, but is present at site; reference in document file is missing or inaccurate
**SS**	Document present but not superseded
**COMP**	Document present but has not been completed correctly (only relevant to certain documents, e.g. faxback document, delegation log, consent forms). The grading will depend on the nature of the completion error
**MISS**	Document is completely absent from site, but this is not indicative of a more serious compliance issue (i.e. the problem is solved by providing the site with the document, or by the site finding the document if they had lost it, or creating the document in the case of patient logs), although the absence of the document in the first place may or may not be serious in itself
**EXIST**	Document is completely absent from site, and is found not to exist (only applicable to documents not provided by CTU, and documents that cannot be recreated retrospectively). The absence of the document may or may not be serious
**DEV**	Regulatory, protocol or other compliance issue, aside from documentation. This category is used for all cases where documentation is **present** and confirms a deviation. This can include issues identified in source data, temperature logs, CVs and so on. It is distinct from the other categories above, which all refer to document being missing, badly organised or badly completed.
**CAU/EFF**	This is used for findings which are possibly best thought of as causes or effects of other findings. They are likely to be subjective, and harder to prove, or are conclusions reached from other evidence. Examples include 'Lack of PI oversight', or 'Site not aware of latest safety developments'. They will not be graded in this document, but may be listed in order to confirm the lack of grading, because they may be recorded in monitoring visit reports.

1A. INVESTIGATOR SITE FILE (ISF) GENERAL			
	**Other**	**Major**	**Critical**
**Site File folders**		**MISS:** part or all of the site file cannot be located, but is found at a later date (NB assume EXIST finding until folders found)	**EXIST:** part or all of the site file cannot be located because it does not exist (NB assume EXIST finding until folders found)
**Site file contents – overall** (see Table 1B for individual documents)		**DEV:** most essential documents missing from site file possibly showing that site staff are not fully aware of the trial and its procedures	

1B. INVESTIGATOR SITE FILE (ISF) AND PHARMACY FILE (PF) CONTENTS

Please note, where essential documents must be maintained in both the ISF and PF and have the same gradings, they will appear in the same row; where the gradings are different, they will appear in different rows. If a document is listed as ISF only a finding relating to it is found in the PF, this can be assumed to be Other.

	Other	Major	Critical
**Current Investigators Brochure (ISF or PF)**	**REF**	**MISS**<br>NB for some findings, it is not clear whether the site might have held the IB electronically. If the site was not asked about this, an Other grade is given, based on benefit of doubt.	
**Superseded IB (ISF or PF)**	**REF**<br>**SS**<br>**MISS:** less than or equal to 2 previous IB versions missing	**MISS:** more than 2 previous IB versions missing and confirmed not available anywhere at site (if this is not confirmed, i.e. for a finding on an old report, this will remain as Other)	
**IB fax back reply (any versions; ISF or PF)**	**COMP:** completed but with errors, or not completed at all (likely to be known already at CTU), but related IB version present at site<br><br>**MISS or REF:** with related IB version present		
**Current SmPC (ISF or PF)**	**MISS**<br>**REF**		
**Superseded SmPC (ISF or PF)**	**REF**<br>**SS**<br>**MISS**		
**Current Protocol (ISF or PF)**	**REF**		**MISS**<br><br>(Confirmed not available anywhere at site; if this is not confirmed, i.e. for a finding on an old report, this will remain as Other)

1B. INVESTIGATOR SITE FILE (ISF) AND PHARMACY FILE (PF) CONTENTS

Please note, where essential documents must be maintained in both the ISF and PF and have the same gradings, they will appear in the same row; where the gradings are different, they will appear in different rows. If a document is listed as ISF only a finding relating to it is found in the PF, this can be assumed to be Other.

	Other	Major	Critical
**Superseded protocol versions (ISF or PF)**	**REF**<br>**SS**<br>**MISS:** one previous protocol version not available at site	**MISS:** more than one previous protocol version not available at site and confirmed not available anywhere at site (if this is not confirmed, i.e. for a finding on an old report, this will remain as Other)	
**Fax back reply for protocol receipt – current protocol version (ISF only)**	**COMP:** completed but with errors, or not completed at all (likely to be known already at CTU), but current protocol version present at site<br>**MISS or REF:** with current protocol present	**COMP:** completed but with errors, or not completed at all (likely to be known already at CTU), and current protocol version not present at site<br>**MISS or REF:** with current protocol missing	
**Fax back reply for protocol receipt – superseded protocol versions (ISF only)**	**COMP:** completed but with errors or not completed at all		
**Current CRFs (samples only) (ISF only)**	**REF:** Blank version of CRFs not present in ISF but site is using the most recent version to complete data	**MISS:** Blank version of CRFs (where changes reflect changes to general data) not present in ISF and site is using incorrect version (likely to be already known at CTU)	**MISS:** Blank version of CRFs (where changes reflect important safety changes/endpoint data) not present in ISF and site is using incorrect version (likely to be already known at CTU)
**Superseded CRFs (ISF only)**	**SS**<br>**REF**<br>**MISS**		
**CRF completion guidelines (current and superseded) (ISF only)**	**REF**<br>**SS**<br>**MISS**		

**1B. INVESTIGATOR SITE FILE (ISF) AND PHARMACY FILE (PF) CONTENTS**<br>Please note, where essential documents must be maintained in both the ISF and PF and have the same gradings, they will appear in the same row; where the gradings are different, they will appear in different rows. If a document is listed as ISF only a finding relating to it is found in the PF, this can be assumed to be Other.			
	**Other**	**Major**	**Critical**
**Participant information sheet (PIS), consent forms and GP letter**<br>**(ISF only)**	**REF:** Blank copies of the PIS and consent form not filed in ISF, but site is using the most recent version in the clinic<br>**COMP:** Documents present in ISF but not on headed paper.	**MISS:** Blank copies of the PIS and consent form not filed in ISF and site is not using most recent version in the clinic, however the most recent version **had no changes to safety information.**	**MISS:** PIS and consent form not filed in ISF and site is not using most recent version in the clinic, where the newer version **has important safety update information.**
**Superseded PIS, Consent forms and GP letter**<br>**(ISF only)**	**SS**<br>**MISS**<br>**REF**		
**Clinical Trial Agreement**<br>**(ISF only)**	**REF:** Copies of appropriate agreements could not be located in ISF or non-signed copy only is in ISF (but completed agreement will exist at CTU)		
**Insurance statement/MRC Statement on indemnity**<br>**(ISF only)**	**REF**<br>**MISS**		
**Ethics approval documentation**<br>**(ISF only)**	**REF**		
**Regulatory approval documentation**<br>**(ISF only)**	**REF**		

**1B. INVESTIGATOR SITE FILE (ISF) AND PHARMACY FILE (PF) CONTENTS**<br>Please note, where essential documents must be maintained in both the ISF and PF and have the same gradings, they will appear in the same row; where the gradings are different, they will appear in different rows. If a document is listed as ISF only a finding relating to it is found in the PF, this can be assumed to be Other.			
	**Other**	**Major**	**Critical**
**R & D Approval documentation: original approval and substantial amendments**<br>**(ISF only)**	**REF:** all required R&D approval letters available, but not appropriately filed.	**MISS:** 1 or more R&D approval letters cannot be found at the time of the meeting, but are provided at a later date (NB assume Critical until document is provided)	**EXIST:** 1 or more R&D approval letters cannot be found at the time of the meeting, and are never located, indicating that R&D approval has not been granted (NB assume this grading until documents provided)<br><br>**DEV:** the site confirms that, for 1 or more protocol amendments or original approval, no R&D approval has been granted.
**R & D Approval documentation: non-substantial amendments**<br>**(ISF only)**	**REF:** evidence is available that R&D has been notified of non-substantial amendments, but appropriate documents have not been filed<br><br>**MISS**		
**Investigator Statement**<br>**(PI only)**<br>**(ISF only)**	**REF:** Investigator statement not filed in ISF but this has been completed (available at site or CTU)<br><br>**COMP:** Investigator agreement has been completed but with errors (not previously noted at CTU)		
**Investigators CVs**<br>**(PI only)**<br>**(ISF only)**	**REF**<br>**MISS:** appropriate CVs not present, or present but not current		**DEV:** CV present but indicates that investigator is not appropriately trained for role (not previously noted at CTU)
**Investigator GCP**<br>**(PI only)**<br>**(ISF only)**	**REF**<br><br>**MISS:** appropriate GCP training not present, or present but not current		**MISS:** No evidence that site staff are appropriately trained in GCP.

1B. INVESTIGATOR SITE FILE (ISF) AND PHARMACY FILE (PF) CONTENTS

Please note, where essential documents must be maintained in both the ISF and PF and have the same gradings, they will appear in the same row; where the gradings are different, they will appear in different rows. If a document is listed as ISF only a finding relating to it is found in the PF, this can be assumed to be Other.

	Other	Major	Critical
**Current delegation log template**<br>**(ISF only)**	**REF:** current template is available (blank or completed) but not correctly referenced in the ISF<br><br>**MISS:** current template cannot be found, and site is not using the current version		
**Superseded delegation log templates**<br>**(ISF only)**	**REF**<br>**MISS**<br>**SS**		
**Completed delegation log**<br>**(ISF only)**<br>(presence of completed document only; for completion errors and possible compliance issues see table 1C below)	**REF**	**MISS**	
**ISF staff training log (trial training)**<br>**(ISF only)**	**REF**<br>**COMP:** Staff trained, but training not documented.<br><br>**MISS:** no training log available	**DEV:** One or two staff not trained for the jobs they are performing.	
		**DEV:** Out of date training logs/no evidence of timely essential training of staff.	
		**CAU/EFF:** Information not passed on from PI to one or two members of staff.	
		**DEV:** Essential training not given to one or two members of staff via PI.	
**Previous Monitoring Reports**<br>**(ISF and PF)**	**REF**<br>**MISS**		
**Monitoring log**<br>**(ISF and PF)**	**COMP**<br>**REF**<br>**MISS**		

1B. INVESTIGATOR SITE FILE (ISF) AND PHARMACY FILE (PF) CONTENTS<br>Please note, where essential documents must be maintained in both the ISF and PF and have the same gradings, they will appear in the same row; where the gradings are different, they will appear in different rows. If a document is listed as ISF only a finding relating to it is found in the PF, this can be assumed to be Other.			
	**Other**	**Major**	**Critical**
**Site Master File Self-Assessment Form (all versions)**<br>**(ISF only)**	**COMP**<br>**REF**<br>**MISS**		
**Laboratory ranges**<br>**(ISF only)**	**MISS:** lab ranges not available, or available but very out of date<br>**REF**<br>**COMP:** Lab normal ranges not signed and dated by lab personnel.		
**Lab accreditation documentation**<br>**(ISF only)**	**REF**	**MISS**	**EXIST:** Lab not accredited.<br>**DEV:** documents show that lab has never been or is or is no longer accredited
**Patient screening log**<br>**(ISF only)**	**COMP:** Screening register not up to date and accurate.<br>**REF**<br>**MISS**	**EXIST or DEV:** based on the evidence available, the site is not screening any patients for the trial	
**Reference to source data**	**MISS**<br>**REF**		
**Randomisation confirmations**<br>**(ISF and PF)**	**REF**<br>**MISS:** one or more confirmations missing but no problems noted as a consequence (e.g. delays in patients receiving medication from pharmacy)	**MISS:** one or more confirmations missing and problems noted as a consequence (e.g. delays in patients receiving medication from pharmacy)	
**Patient Allocation list**<br>**(ISF and PF)**	**COMP:** Trial number allocation register not up to date and accurate.<br>**REF**	**MISS:** No patient log or process in place to correctly identifying patients using trial ID number.	
**All Annual SUSAR Reports/DSURs**<br>**(ISF and PF)**	**REF**<br>**MISS**		

**1B. INVESTIGATOR SITE FILE (ISF) AND PHARMACY FILE (PF) CONTENTS**<br>Please note, where essential documents must be maintained in both the ISF and PF and have the same gradings, they will appear in the same row; where the gradings are different, they will appear in different rows. If a document is listed as ISF only a finding relating to it is found in the PF, this can be assumed to be Other.			
	**Other**	**Major**	**Critical**
**Annual Safety Report faxbacks (ISF only)**	**REF**<br>**MISS:** one or more completed faxbacks missing at site and CTU<br>**COMP:** completion errors not previously noted at CTU		
**SAE completion guidelines (all versions)**<br>**(ISF only)**	**REF**<br>**MISS**<br>**SS**		
**List of SAEs for the site (ISF only)**	**COMP REF**<br>**MISS**		
**Completed SAE reports**<br>**(ISF only)**<br>This refers to SAEs known already at the CTU. For findings relating to unreported SAEs, including completed SAE forms not submitted, see Table 4c	**REF:** one or more SAE forms held outside the ISF and not properly referenced.<br><br>**MISS**		
**Unblinding procedures**<br>**(ISF and PF)**	**REF**<br>**SS**	**MISS**	
**All other documents (including table of contents)**<br>**(ISF and PF)**	**REF**<br>**MISS**<br>**SS**<br>**COMP**		
**PHARMACY-SPECIFIC DOCUMENTS (NB this section relates to template pharmacy documents. For completion errors and other pharmacy issues, including any documents relating to trial participants, please see Table 3)**			
**Sample label**	**REF**<br>**SS**<br>**MISS:** sample not present in PF but no problems noted with labels used for trial patients	**MISS:** sample not present in PF and noted problems with labels used for trial patients	

1B. INVESTIGATOR SITE FILE (ISF) AND PHARMACY FILE (PF) CONTENTS<br>Please note, where essential documents must be maintained in both the ISF and PF and have the same gradings, they will appear in the same row; where the gradings are different, they will appear in different rows. If a document is listed as ISF only a finding relating to it is found in the PF, this can be assumed to be Other.			
	**Other**	**Major**	**Critical**
**Sample label sheets**	**REF**<br>**SS**<br>**MISS:** sample label sheet not present in PF but no problems noted with sheets used for trial patients	**MISS:** sample label sheet not present in PF and noted problems with sheets used for trial patients	
**Current pharmacy information sheet**	**REF**	**MISS**: Pharmacy information sheet missing and no additional documents (local SOPs/information sheets) present which describe pharmacy procedures.<br><br>NB for some findings, it is not clear whether the site might have held the pharmacy information sheet electronically. If the site was not asked about this, an Other grade is given, based on benefit of doubt.	
**Superseded pharmacy information sheet(s)**	**REF**<br>**MISS**<br>**SS**		
**Drug order form template (all versions)**	**REF**<br>**MISS:** template missing, but no errors noted with drug order forms used<br>**SS**	**MISS:** template missing and noted errors with drug order forms used	
**Pharmacy site file self-assessment form (all versions)**	**REF**<br>**SS**<br>**COMP or MISS:** completed with errors, not completed or missing altogether, but no significant documents missing from PF		
**Drug shipment record template (all versions)**	**REF**<br>**SS**<br>**MISS**		

1B. INVESTIGATOR SITE FILE (ISF) AND PHARMACY FILE (PF) CONTENTS<br>Please note, where essential documents must be maintained in both the ISF and PF and have the same gradings, they will appear in the same row; where the gradings are different, they will appear in different rows. If a document is listed as ISF only a finding relating to it is found in the PF, this can be assumed to be Other.			
	**Other**	**Major**	**Critical**
**Template destruction log (all versions)**	**REF**<br>**SS**<br>**MISS:** no template available, but no problems noted relating to drug destruction	**MISS:** no template available, site not destroying stock appropriately	
**Template accountability log (all versions)**	**REF**<br>**SS**<br>**MISS**		
**Local pharmacy SOPs**	**REF**<br>**SS**	**MISS:** inadequate local SOPs to be able to comply with trial procedures	**DEV:** local SOPs directly contradict the protocol or GCP
**Pharmacist CVs**	[No suggested grades, but no cases in TEMPER]		
**Pharmacist GCP certificates**	[No suggested grades, but no cases in TEMPER]		
**Pharmacist training log**	[No suggested grades, but no cases in TEMPER]		

1C. DELEGATION LOG (see Table 1B above for findings relating to absence/presence of log)			
	**Other**	**Major**	**Critical**
**Header details**	Header details (excluding PI details) are incorrect or missing		Principal Investigator entry has been completed by someone who is not (and never has been) the PI.
**Site Staff not listed**	Site staff not listed on delegation log, but clearly performing important trial duties (e.g. consenting patients); PI confirms delegation of duties retrospectively; **3 or less staff members affected.**<br><br>Staff are listed on the log twice<br><br>Staff known to work on non-critical aspects of the trial (e.g. CRF completion) are not listed on the delegation log but can be added later.<br><br>Pharmacy staff are not listed on the ISF log, and there is no pharmacy-specific log, but can be added later.	Site staff not listed on delegation log, but clearly performing important trial duties (e.g. consenting patients); PI confirms delegation of duties retrospectively; **4 or more staff members affected**.	PI is not aware of a member of staff who is carrying out trial specific tasks.
**Study Role**	Study roles missing or inaccurate		
**Signatures/Initials**	A few signatures and/or initials missing on the log		
**Delegated tasks**	Delegated tasks not entered for members of staff, or do not correspond to roles (with no safety implications)	Delegated tasks not entered for members of staff, or do not correspond to roles, with safety implications (e.g. staff taking consent when not delegated to do so)	

**PI authorisation**	PI has not signed to authorised delegation to a members of staff [see sections above for staff the PI is not aware of]		
**End dates**	End dates not added for staff who have stopped working on the trial		

2. INFORMED CONSENT			
	**Other**	**Major**	**Critical**
**2A. Missing Forms**			
**Consent forms unavailable but alternate evidence available**	**REF**<br>**MISS:** consent forms could not be located at visit but are provided in response to monitoring report<br><br>Consent form for registration (not randomisation) or substudy enrolment (one case only for Other) cannot be found, but evidence available that patient was consented.	Main consent form cannot be found, but evidence available that the patient was consented<br><br>More than one consent form for substudy registration cannot be found.	If > 10% or >2 (whichever is larger)consent forms cannot be found, even if there is evidence that the patient was consented, upgrade group of findings to critical
**Consent forms unavailable and no supporting evidence of patient consenting available**		Consent form cannot be provided, with no evidence of consent elsewhere, e.g. in patient notes – only affecting substudy or other non-main consent.	Main consent form cannot be found and no other evidence of consent to the trial.

2B. WHOLE FORM ASSESSMENT			
**Poor consent form completion – general**			If a site has Major findings in more than two different areas of consent form completion, this should be upgraded to one Critical.
**Consent Form Version –minor changes only**	Incorrect version used	Incorrect version used on a multiple occasions, >1 month after receiving local approval, upgrade group of findings to major	
**Consent Form Version – including altered safety information**	Incorrect version used within 1 month of gaining local approval	Incorrect version used >1month after local approval	If > 10% or >2 (whichever is larger) patients were consented using incorrect version, >1 month after receiving local approval, upgrade group of findings to critical
**Headed paper**	Consent form not presented on local headed paper		

**ID/Centre number**	ID/centre number not added after randomisation<br><br>ID not required to be added on consent form template, but not possible to ascertain which patient it relates to		
**Corrections**	Correction made to non-critical section (e.g. patient ID) without initial and date of who made the change	Correction made to critical section (e.g. statement boxes, signatures, dates) without initial and date of who made the change<br><br>**NB** in general, benefit of doubt was given by ERC in these cases and they were mostly graded Other. A collection of all cases was reviewed by the ERC in June 2016.	Strong evidence that Investigator has backdated consent forms or made some other fraudulent entry/change.
**2C. PIS DETAILS ENTRY**			
**PIS version and date not added, incomplete or invalid**	PIS version number missing from consent form, but there is evidence patient was given correct PIS (filed in notes or documented)	PIS version number missing from consent and no way of knowing if they read the correct PIS.	Not possible to determine which PIS version many patients read, upgrade group of findings to critical (definition of 'many' decided on ad hoc basis in this case)
**Incorrect PIS version where change since previous version did not involve updated safety information**	Incorrect version of the consent form used on few occasions but the amendment did not update safety information	Incorrect version of consent/PIS used for many (definition of 'many' decided on ad hoc basis) patients after new version was approved, upgrade group of findings to major<br>(**NB** can be Other if site takes corrective action independently and in relatively short time; ERC to review on case-by-case basis)	

**Incorrect PIS version where change since previous version involved updated safety information or important information about trial changes**	Incorrect version of the consent form used within 1 month of local approval being granted	Incorrect version of the consent form used, > 1 month after approval, where safety information was updated<br><br>**NB** where the dates are not clear, ERC sometimes has given benefit of doubt. Final review of uncertain cases conducted June 2016.	Incorrect version of consent/PIS used for many patients ('many' definition decided ad hoc) after new version was approved, upgrade group of findings to critical
**2D. CONSENT FORM STATEMENTS**			
**Initials provided in each mandatory box**	Patient has ticked instead of initialling.		
**Completion of all mandatory boxes**		Not all mandatory boxes have been completed, but patients have nonetheless been randomised.	
**Optional consent boxes**		Patients' optional consents have been misreported to CTU on CRFs or at randomisation	Patient has not consented to an optional consent statement, but a trial procedure has been carried out regardless.
**2E. SIGNATURES AND DATES**			
**Signatures present**<br><br>**NB** for issues to do with authority to consent, see delegation log section, above.	Signatures present but incorrectly placed (e.g. in 'Print name' space)	Signatures are present, but the patient initials on the rest of the form do not match the patient's signature [only Major if certain]	Signatures are present, but do not match patient's entries (e.g. in handwriting) elsewhere on the form

**Dates present**	Dates entered are invalid and not corrected, but the error is obvious (e.g. previous year entered on a consent completed in January of a given year)	Dates not entered by patient and/or clinician (**NB** ERC can grade Other if the site provides a satisfactory explanation, e.g. the patient reconsented before randomisation)<br><br>Dates are present but patient's and clinician's are different (**NB** this can be Other if the site provides an adequate explanation, e.g. evidence that one date is a typo, or the patient pre-filled in a form at home. Generally, where the clinician's date is before the patient's, this is usually Major)	Dates are present but either of them is after the date of randomisation (this could be downgraded if it is found to be a reconsent for any reason, and an initial consent to randomisation exists). **NB** this may overlap with grading of treatment procedure timing findings; see section 4C, below. Only one grade should be applied in each case.<br><br>Date of consent is different for patient and clinician on a number of forms, upgrade to critical
**Clinician or patient signature missing**	Clinician signature missing on non-main consent form (substudy, registration).	Patient and/or clinician signature missing from main consent form.	If consent has not been signed correctly for >3 main consents then this group of findings should be upgraded.
**Witness not present when required**		No indication if witness was present/absent if illiterate.	If there was no indication of witness being present/absent if illiterate for many patients then this group of findings should be upgraded to critical.
**2F. RECONSENT**			
**Pt not re-consent to new versions where <u>Safety info is updated</u>**		- Patient not re-consented as appropriate (Safety information)	Site missed at least one required reconsent on more than 50% of the occasions (protocol amendments) where this was required.<br><br>(**NB** this threshold was chosen as a compromise, because sometimes establishing the number of reconsents required is difficult. The final TEMPER upgrades are based on this threshold.)
**Delay re-consenting patients**		Excessive delay (>6 months)in re-consenting patients to amendment	If there was an excessive delay in re-consenting many patients to amended consent forms, this group of findings should be upgraded

3. PHARMACY COMPLIANCE (for generic and template pharmacy documents, please see Table 1B)			
	**Other**	**Major**	**Critical**
**3A. Accountability Logs/ Dispensing & returns records/ Destruction documentation**			
**Site using own template documentation**	Site using their own template accountability/dispensing or destruction logs.		
**Accountability log/ Dispensing &returns records/ Destruction documentation completion**	All documentation errors in dispensing, accountability and destruction to be graded other, so long as there is additional evidence (prescriptions) to suggest that patients received the correct dose of the correct drug.		
**3D. IMP Dispensing errors**			
**Incorrect dose dispensed** (NB if noted in pharmacy as well as in source data, only one finding results)		Incorrect dose of IMP prescribed/administered to patient (No safety concerns)	Incorrect dose of IMP prescribed/administered to patient (Safety Issue)
**Inappropriate stock dispensed**	Drug given from ward stock instead of trial stock to trial patient	Trial stock given to patient on another trial.	Participants given wrong treatment (arm)/inappropriate drug administration without the site being aware of the error<br>IMP given to patients who were not on the trial<br>Expired IMP dispensed to participants<br>Evidence that patients were dispensed the wrong bottle number of blinded IMP (if applicable).
**3F. IMP Stock**			

3. PHARMACY COMPLIANCE (for generic and template pharmacy documents, please see Table 1B)			
	**Other**	**Major**	**Critical**
**IMP Storage**<br><br>**[Trial specific due to differences in dispensing systems used]**	Expired IMP stored alongside in date IMP, but dispensation controlled by IVRS. (Graded other as dispensed using IVRS which takes into account expiry date automatically)	Expired open-label IMP stored alongside in date stock without being quarantined<br><br>Giving other staff members who are not authorized, access to pharmacy records and/or trial drug<br><br>No IMP in stock despite trial being open to recruitment and/or still having patients on trial.	IMP stored incorrectly, including:<br>- stored on floor<br>- unlabelled<br>- in inappropriate fridge (i.e. with food)
**3G. Temperature Checks**			
**Temperature logs**	Temperature records not on file, but are available.	IMP storage temperature not recorded every working day	IMP storage temperature not checked and/or recorded at all
**Temperature deviations**	Temperature found to have gone out of range, but drug would still be fit for use.	Temperature deviated to such an extent that the drug would no longer be fit for use, however it is proven that none of the IMP affected has been prescribed	Temperature deviated to such an extent that the drug would no longer be fit for use, and it is found that the IMP affected has been prescribed

**4A. SOURCE DATA AND CRFS AVAILABLE**<br>**(NB for any confirmed deviations – e.g. eligibility criteria not verifiable in source documents because patient is confirmed ineligible – see Table 4C. deviations identified in source data)**			
	**Other**	**Major**	**Critical**
**Source data review – general**			If a site has Major findings in more than two different areas of source data review, this should be upgraded to one Critical.
**Source data – available for all submitted data (i.e. data on CRFs present or absent in source, for whole visits, whole assessments or single data points)**	Source data not available to be able to verify data unrelated to eligibility or endpoints. Always graded Other initially.	Upgrade to Major if site are unable to provide source data to verify patient eligibility or endpoint data or important safety information following completion of actions on report.	
**Completed CRFs**	CRFs known to be completed (i.e. received at CTU) not available at site , for example because site not routinely keeping copies<br><br>Many CRFs completed but not sent to CTU<br><br>Updated CRFs not submitted to CTU; non-critical data		

**4B. CRF COMPLETION**<br>**(NB where a CRF completion error indicates a protocol violation, e.g. patient is not eligible, see Table 4C below for gradings relating to these.)**			
	**Other**	**Major**	**Critical**
**CRF completion**			
**Data on CRF does not match source data**<br>(NB for cases where there is no source data – even regarding single data points – see Table 4A)	Data on CRFs (includes toxicity data; SAE data to be treated on a case-by-case basis) does not match source data, but the incorrect data does not relate to endpoints or patient safety monitoring.		

4B. CRF COMPLETION (NB where a CRF completion error indicates a protocol violation, e.g. patient is not eligible, see Table 4C below for gradings relating to these.)			
	**Other**	**Major**	**Critical**
**Data return rate**	Excessive delay in sending CRFs for data entry.		

4C. DEVIATIONS IDENTIFIED IN SOURCE DATA			
	**Other**	**Major**	**Critical**
**Eligibility and Randomisation**			
**Patient found to be ineligible**<br><br>**(includes items not reported at randomisation, items reported incorrectly and tests not done)**	Certain cases of ineligible patients can be Other, on TMT advice<br><br>Patient ineligible but no error at site – randomised 'in good faith' based on information available (case-by-case review as to whether this argument applies)	Participants enrolled in the study, but not meeting eligibility criteria (not safety issue – assess on a case by case basis)	Many (definition decided on ad hoc basis) participant(s) enrolled in the study, but not meeting eligibility criteria (not safety issue – assess on case by case basis), upgrade grouped findings to critical<br><br>Any participant(s) enrolled in the study, but not meeting eligibility criteria (safety issue – assess on case by case basis)
**Randomisation data incorrect**	"Insignificant baseline data" reported incorrectly; patient still meets eligibility criteria	"Significant baseline data" reported incorrectly; patient still meets eligibility criteria	
	See trial specific definitions of significant & insignificant baseline data, for TEMPER purposes; these are documented on annotated baseline CRFs stored alongside Appendix C. Highlighted fields on these are the 'significant' data according to the gradings given above.		
**Patient randomised between wrong arms**			Data provided at randomisation was incorrect; patient eligible for trial in general but not for allocated treatment

4C. DEVIATIONS IDENTIFIED IN SOURCE DATA			
	**Other**	**Major**	**Critical**
**Timing of first trial procedures and intervention administration**<br><br>(**NB** if this is noted in pharmacy and/or consent form review as well as in source data, only one finding results)			Study procedures done before consent taken (**NB** this may overlap with the consent form gradings above, see grades for consent taken after randomisation, section 2E. Only one grade should be applied in each case)<br><br>Participant screened for the trial before MREC/MHRA /R&D/sponsor approval was obtained.<br><br>Post-randomisation/registration procedures (including intervention administration) begun before randomisation/registration<br><br>Intervention administered prior to approval from CTU, or before safe for patient to receive it
**Follow up, treatment and trial procedures**			
**Regularity of patient follow up**	Patients not followed up with regularity mandated by protocol, but no impact on safety of participants or collection of endpoint data (i.e. in time-to-event trial)<br><br>Patient withdrawn from data collection without reason for doing so being documented	Patients not followed up with regularity mandated by protocol, and possible impact on impact on safety of participants or collection of endpoint data (i.e. in time-to-event trial) – whether it constitutes Major finding decided on case-by-case basis.<br><br>>10% of patients found to be withdrawn from data collection without reason for doing so being documented, upgrade to major	

4C. DEVIATIONS IDENTIFIED IN SOURCE DATA			
	**Other**	**Major**	**Critical**
**Unreported endpoint data**	Secondary endpoints not reported on CRFs at all/in timely manner.<br><br>Primary endpoint(s) not reported at all but less than a year since the event occurred.<br><br>Primary endpoint not reported at all but subjectively defined.	Primary endpoint not reported for over a year since it occurred and clear-cut case (e.g. patient died).	Clear-cut primary endpoints not reported, for more than one patient at a site, or more than one for a single patient, for over a year since it/they occurred.
**Protocol specified tests/assessments missed (post-trial entry)**	Protocol specified lab tests/investigations/assessments not performed. Does not impact on safety (decided on case-by-case basis)	Unnecessary trial procedures performed (e.g. unnecessary tests or scans performed, or patients followed up too often) on a few occasions (decided on case-by-case basis)	If many protocol specified lab tests/investigations not affecting patient safety not performed, upgrade grouped findings to critical.<br><br>Any protocol specified lab tests/investigations/assessments not performed that impact participant safety.
**Treatment not following protocol**<br>(NB if noted in pharmacy as well as in source data, only one finding results)	Treatment breaks outside of those permitted in the protocol	Protocol dose changes not being followed (e.g. if patient experiences toxicity or change in weight then dose should be decreased according to protocol)<br><br>Incorrect dose of IMP prescribed/administered to patient (No safety concerns)	Incorrect dose of IMP prescribed/administered to patient (Safety Issue)
**Contraindicated medication administered**		Recommendation contraindication (not specifically in eligibility criteria) not followed at site.	Medication contraindicated in trial eligibility criteria/similar given to trial patient.
**Clinical assessments showing results out of normal ranges**	Reports not signed off by trial clinician in timely fashion.<br><br>Lab reports not signed off, but evidence that abnormal lab results have been actioned.	If many (definition decided on ad hoc basis) lab reports are not signed off by trial clinician in timely fashion: upgrade group of findings to Major.	
**Unblinding**		Site unblinding patient(s) unnecessarily	Sites not unblinding when necessary to protect patient safety

4C. DEVIATIONS IDENTIFIED IN SOURCE DATA			
	**Other**	**Major**	**Critical**
**SAE Reporting**			
**SAEs not reported** (including where SAE form completed but not submitted to CTU)		SAEs not reported according to the required reporting procedures. Including SAEs required by the protocol (e.g. notable events)	If a site is found to have >1 patient with an unreported SAE, or 1 patient >2 unreported SAE, upgrade group of findings to critical (in cases where both these conditions satisfied, one Critical applied only).<br><br>Any SUSAR not reported to the sponsor.
**SAEs not acted on clinically**		SAEs/Notable events discovered but not acted upon clinically.<br><br>SAEs not acted on (i.e. missed altogether and not treated according to protocol/standard of care)	If many SAEs/Notable events discovered but not acted upon clinically upgrade group of findings
**Delay in reporting SAEs**	Trial team reported SAE within 1 day of becoming aware; however delay in research team being notified of SAE.	SAE reported > 1 day after the research team at site became aware of the event.	If more than 3 or more SAEs are reported late (> 1 day of site being aware of event) then this group of findings should be upgraded to Critical.

5. GENERAL:			
	**Other**	**Major**	**Critical**
**Site conduct**		Test kits stored incorrectly or used beyond their expiry date<br><br>Site staff appear to be obstructive towards the organisation and the visit.	Confidentiality: Participant files/data not locked away correctly
