# Supplementary material for: Triggered or routine site monitoring visits for randomised controlled trials: results of TEMPER, a prospective, matched-pair study
Source: Clin Trials. 2018 Aug 22;15(6):600–9. doi: 10.1177/1740774518793379 (PMC6236642; doi:10.1177/1740774518793379)
Supplement: 793379_supp_mat_4 – Supplemental material for Triggered or routine site monitoring visits for randomised controlled trials: results of TEMPER, a prospective, matched-pair study [file 793379_supp_mat_4.pdf]

Pre-visit questionnaires

Once sites were selected for a visit, but before the visit took place, the trial teams at the site, and at the Clinical Trials Unit, were asked to complete short questionnaires. These addressed issues that the TEMPER team believed might potentially predict on-site findings

Site pre-visit questionnaire

The main contact person at the site was asked to complete the questionnaire, on which the following questions were asked:

- Total number of active trials (recruiting or in follow-up), including cancer trials, open at the hospital
- Total number of active cancer trials open at hospital

For each of: Principal Investigator, Research nurse, Trial Coordinator, Data manager, Pharmacist:

- Number of trials worked on
- Who attends multidisciplinary team meetings?
- Who is responsible for training new staff?
- Who completes the trial CRFs?
- Who reviews trial CRFs?
- Who carries out the pre-screening activities for enrolment of potential patients into the trial?
- Who discusses the trial with the patients and answers any questions during the informed consent process?

Clinical Trials Unit pre-visit questionnaire

The Trials Unit team were asked to discuss the following questions, and grade the site on each question on a 5 point scale (Excellent, Good, Satisfactory, Poor, Very Poor)

- Overall impression of resources at site
- Overall impression of site performance
- Speed of response to communication (email, phone)
- Quality of communication from site (email, phone)
- Speed of data query response
- Quality of data query response
