# Supplementary material for: Triggered or routine site monitoring visits for randomised controlled trials: results of TEMPER, a prospective, matched-pair study
Source: Clin Trials. 2018 Aug 22;15(6):600–9. doi: 10.1177/1740774518793379 (PMC6236642; doi:10.1177/1740774518793379)
Supplement: 793379_supp_mat_5 – Supplemental material for Triggered or routine site monitoring visits for randomised controlled trials: results of TEMPER, a prospective, matched-pair study [file 793379_supp_mat_5.pdf]

Supplementary Tables

Table S1: Results by trial

	Any Major or Critical Finding N (%)		Any Major or Critical finding excluding re-consent N (%)		Any Major or Critical finding excluding all consent N (%)		Any Critical Finding N (%)		Any Critical finding excluding re-consent N (%)		Any Critical finding excluding all consent N (%)	
	T*	U*	T	U	T	U	T	U	T	U	T	U
Trial 1	16 (100)	14 (88)	15 (94)	9 (56)	12 (75)	9 (56)	8 (50)	3 (19)	5 (31)	2 (13)	4 (25)	2 (13)
Trial 2	10 (83)	7 (58)	10 (83)	7 (58)	9 (75)	6 (50)	4 (33)	3 (25)	4 (33)	3 (25)	3 (25)	3 (25)
Trial 3	11 (79)	13 (93)	11 (79)	9 (64)	8 (57)	4 (29)	3 (21)	2 (14)	3 (21)	0 (0)	3 (21)	0 (0)

***T= Triggered Visit, U=Untriggered visit**

Table S2: Summary of Critical findings observed at TEMPER monitoring visits.

Monitoring Report Section	At presentation [a] or upgrade? [b]	Details	No. findings paired visits	No. findings HR [c] visits
Consent form review	At presentation	Multiple issues with a patient's consent.	2	0
Consent form review	At presentation	No valid consent prior to randomisation	1	0
Consent form review	Upgrade	Upgrade due to multiple Major findings on several different aspects of consent form completion.	3	0
Consent form review	Upgrade	Upgrade due to failure to re-consent multiple patients.	8	1
Pharmacy	At presentation	Patient in double-blind trial given incorrect treatment bottle.	1	1
Case Report Form review/ source data verification	At presentation	Patient randomised before eligibility confirmed and consent provided.	1	0
Case Report Form review/ source data verification	At presentation	Patient found to be ineligible, with implications for patient safety.	6	2
Case Report Form review/ source data verification	Upgrade	Upgrade due to multiple unreported trial outcome data.	4	0
Case Report Form review/ source data verification	Upgrade	Upgrade due to multiple unreported Serious Adverse Events.	4	0

[a] 'At presentation' refers to findings attracting a Major or Critical grade on their own.

[b] 'Upgrade only' refers to groups of findings from the same visit that, collectively, attract a higher grade (for example, a series of Major findings at the same site could, in some circumstances, be upgraded to one Critical finding).

[c] HR = High Recruiter

Table S3: Assessing the prognostic value of individual triggers – binary outcomes

Triggers fired at time of site selection		Proportion of sites meeting each outcome measure											
		≥ 1 Major or Critical finding?		≥ 1 Major or Critical finding, excluding re-consent findings		≥ 1 Major or Critical finding excluding all consent findings		≥ 1 Critical finding?		≥1 Critical finding, excluding re-consent findings		≥ 1 Critical finding, excluding all consent findings	
		N	%	N	%	N	%	N	%	N	%	N	%
**General concern**	No (n=75)	61	81%	51	68%	38	51%	18	24%	13	17%	13	17%
	Yes (n=19)	17	90%	17	90%	15	79%	7	37%	5	26%	3	16%
	**P-value** [a]		P=0.512		P=0.085		**P=0.037**		P=0.261		P=0.513		P=1.000
**CRF return rate**	No (n=57)	46	81%	38	67%	31	54%	14	25%	11	19%	10	18%
	Yes (n=37)	32	87%	30	81%	22	60%	11	30%	7	19%	6	16%
	**P-value** [a]		P=0.579		P=0.160		P=0.675		P=0.637		P=1.000		P=1.000
**Data query rate**	No (n=90)	74	82%	64	71%	49	54%	24	27%	17	19%	15	17%
	Yes (n=4)	4	100%	4	100%	4	100%	1	25%	1	25%	1	25%
	**P-value** [a]		P=1.000		P=0.573		P=0.129		P=1.000		P=0.579		P=0.532
**Data query resolution time**	No (n=59)	45	76%	37	63%	29	49%	13	22%	10	17%	8	14%
	Yes (n=35)	33	94%	31	87%	24	69%	12	34%	8	23%	8	23%
	**P-value** [a]		**P=0.026**		**P=0.008**		P=0.086		P=0.231		P=0.589		P=0.268
**Protocol deviation (2/3 trials)**	No (n=14)	9	64%	9	64%	9	64%	2	14%	2	14%	2	14%
	Yes (n=48)	43	90%	37	77%	30	63%	18	38%	13	27%	11	23%

	**P-value [a]**		**P=0.038**		P=0.488		P=1.000		P=0.192		P=0.484		P=0.715
**Low SAE rate (2/3 trials)**	No (n=53)	41	77%	37	70%	27	51%	11	21%	9	17%	8	15%
	Yes (n=6)	4	67%	4	67%	3	50%	1	1%	1	17%	1	17%
	**P-value [a]**		P=0.620		P=1.000		P=1.000		P=1.000		P=1.000		P=1.000
**High SAE rate (2/3 trials)**	No (n=66)	59	89%	49	74%	37	56%	18	27%	11	17%	10	15%
	Yes (n=1)	0	0%	0	0%	0	0%	0	0%	0	0%	0	0%
	**P-value [a]**		P=0.119		P=0.269		P=0.448		P=1.000		P=1.000		P=1.000
**High recruiting site [b]**	No (n=54)					30	56%					9	17%
	Yes (n=40)					23	58%					7	18%
	**P-value [a]**						P=1.000						P=1.000

[a] p-values from Chi-square test/Fisher's exact test; p values ≤0.05 highlighted

[b] Assessed only with respect to non-consent findings

Table S4: Assessing the prognostic value of individual triggers – continuous outcomes

Triggers fired at time of site selection		Total Major and Critical findings					Total Major + Critical findings, excluding reconsent					Total Major + Critical findings, excluding all consent findings				
		N	Min	Median	Max	P-value [a]	N	Min	Median	Max	p-value [a]	N	Min	Median	Max	p-value [a]
**General concern**	No	75	0	1	33		75	0	1	14		75	0	1	6	
	Yes	19	0	3	24	0.018	19	0	2	7	0.023	19	0	1	3	0.042
**CRF return rate**	No	57	0	2	33		57	0	1	6		57	0	1	6	
	Yes	37	0	1	24	0.590	37	0	1	14	0.454	37	0	1	6	0.846
**Data query rate**	No	90	0	1	33		90	0	1	14		90	0	1	6	
	Yes	4	1	1.5	3	0.807	4	1	1.5	3	0.594	4	1	1	1	0.497
**Data query resolution time**	No	59	0	1	21		59	0	1	7		59	0	0	6	
	Yes	35	0	3	33	0.017	35	0	1	14	0.072	35	0	1	6	0.104
**Protocol deviation (2/3 trials)**	No	14	0	1	5		14	0	1	5		14	0	1	5	
	Yes	48	0	3	33	0.005	48	0	2	14	0.158	48	0	1	6	0.902
**Low SAE rate (2/3 trials)**	No	53	0	1	9		53	0	1	7		53	0	1	6	
	Yes	6	0	1.5	6	0.951	6	0	1.5	6	0.686	6	0	.5	4	0.971
**High SAE rate (2/3 trials)**	No	66	0	1	33		66	0	1	14		66	0	1	6	
	Yes	1	0	0	0	0.119	1	0	0	0	0.269	1	0	0	0	0.448

**High recruiting site** [b]	No											54	0	1	5	
	Yes											40	0	1	6	0.555

[a] p-values from Mann-Whitney test for independent samples; p-values ≤0.05 highlighted

[b] Assessed only with respect to non-consent findings

Table S5: Site staff roles and site visit findings [a]

		≥ 1 Major or Critical finding?		≥ 1 Major or Critical finding, excluding re-consent findings		≥ 1 Major or Critical finding excluding all consent findings		≥ 1 Critical finding?		≥1 Critical finding excluding re-consent findings		≥ 1 Critical finding, excluding all consent findings	
		N	%	N	%	N	%	N	%	N	%	N	%
Number of PI roles (grouped)	<3 (n=8)	6	75%	5	63%	3	38%	1	13%	0	0%	0	0%
	3 (n=20)	18	90%	14	70%	11	55%	4	20%	3	15%	3	15%
	4 (n=22)	19	86%	16	73%	12	55%	6	27%	2	9%	2	9%
	5-6 (n=26)	23	89%	23	89%	19	73%	12	46%	11	42%	9	35%
	P-value [b]		p=0.572		P=0.077		P=0.067		P=0.026		P=0.005		P=0.022
Number of RN roles (grouped)	<=3 (n=16)	15	94%	15	94%	11	69%	6	38%	5	31%	5	31%
	4 (n=17)	17	100%	16	94%	12	71%	7	41%	4	24%	4	24%
	5 (n=20)	17	85%	16	80%	12	60%	8	40%	6	30%	5	25%
	6 (n=21)	15	71%	10	48%	9	43%	2	10%	1	5%	0	0%
	P-value [b]		P=0.018		P<0.001		P=0.077		P=0.063		P=0.08		P=0.021

[a] Possible roles: attended Multi-Disciplinary Team (MDT) meetings, trained new staff, completed CRFs, reviewed CRFs, performed pre-screening activities, and discussed the trial with potential participants.

[b] P-values from Chi-square test for linear trend; p-values < 0.05 highlighted
